# Allelochemical Potential of *Smilax fluminensis* Steud. (Smilacaceae) Leaves: Investigation of the Effects on Germination, Seedling Development and Cellular Alterations

**DOI:** 10.3390/plants15162453

**Published:** 2026-08-12

**Authors:** Lucas Santos Azevedo, Thaís Paula Rodrigues Gonçalves, Gabriela Cristina Ferreira Mota, Mariana Guerra de Aguilar, Lúcia Pinheiro Santos Pimenta, Ana Hortência Fonsêca Castro, Luciana Alves Rodrigues dos Santos Lima

**Affiliations:** 1 Campus Centro-Oeste Dona Lindu, Universidade Federal de São João Del-Rei (UFSJ), Divinopolis 35501-296, MG, Brazil; azevedolucas07@gmail.com (L.S.A.); thaispaula.rgs@gmail.com (T.P.R.G.); acastro@ufsj.edu.br (A.H.F.C.); 2 Departamento de Química, Instituto de Ciências Exatas, Universidade Federal de Minas Gerais (UFMG), Belo Horizonte 31270-901, MG, Brazil; gabrielacfm@ufmg.br (G.C.F.M.); marianag.a9@gmail.com (M.G.d.A.); lpimenta@ufmg.br (L.P.S.P.)

**Keywords:** japicanga, salsaparilha, allelopathy, cytogenotoxicity, antigenotoxicity

## Abstract

Agrochemicals are used worldwide in food production, but their use varies between countries due to the damage observed to nature and human health. Allelopathy is the primary pathway of chemical communication in plants, interfering with biome development through stimulation and/or inhibition mechanisms. Therefore, this study aimed to assess the biological activities of the ethanol extract (EE) and fractions of *S. fluminensis* leaves on monocotyledonous and eudicotyledonous models. The EE was obtained by percolation with ethanol, and the hexane (HEXF), dichloromethane (DCMF), ethyl acetate (EAF), and hydroethanol (HEF) fractions were obtained by liquid–liquid partition. The phytochemical characterization was performed by ^1^H nuclear magnetic resonance (NMR). The allelopathic activity was evaluated on *Allium cepa* (onion) and *Lactuca sativa* (lettuce) seeds. The cytotoxic, genotoxic, and antigenotoxic effects on *A. cepa* meristematic cells were analyzed in vitro. Aliphatic compounds, saponins, and flavonoids derived from quercetin and kaempferol were characterized in the samples. All samples decreased the vigor, germination rate, and germination speed index (GSI) of *A. cepa* seeds. In contrast, they did not alter the vigor and viability of *L. sativa* seeds, but decreased the GSI, except for HEF. The samples inhibited the epicotyl and root growth of *A. cepa* and *L. sativa*, except HEXF, which stimulated the growth of epicotyls (750 µg/mL) and roots (750 and 1000 µg/mL). The cytotoxic assays showed that the EE and HEXF had cytotoxic action at low concentrations, and no sample showed a genotoxic effect. The following samples exhibited an antigenotoxic effect after pretreatment with atrazine (ATZ): EE (125 and 750 µg/mL), HEXF (750 and 1000 µg/mL), DCMF (125, 250, and 1000 µg/mL), EAF (at all tested concentrations), and HEF (125, 250, and 750 µg/mL). Furthermore, the EAF at 125 and 500 µg/mL and HEF at 125 µg/mL demonstrated the potential to reverse genetic damage induced by glyphosate (GLY).

## 1. Introduction

Biotechnological innovations are used to solve problems that afflict humanity, allowing for the development of new technologies for the study and treatment of diseases, as well as the identification of less aggressive alternatives for ecosystems [1,2]. Among the biotechnology applications, the use of plant models allows for development in various areas, such as the production and acquisition of complex metabolites and the provision of study models for the biological activities of chemical compounds at the macro and microscopic levels [3,4]. In this context, numerous studies have confirmed the importance of research related to the conservation of Brazilian Cerrado species for the development of effective strategies for the preservation and management of this biome [5]. These studies have also contributed to research in other areas of knowledge, such as species ecophysiology, germplasm conservation, and seed technology [6,7]. Despite their invaluable potential, plant species from the Brazilian Cerrado remain underexplored and understudied, especially regarding their application for therapeutic and agricultural purposes [8,9].

Agrochemicals are used worldwide in food production, but their use varies between countries due to the damage observed to nature and human health. Classified as a toxic product for the environment, atrazine has been banned in the European Union since 2003, as residues found in cereals, milk, and meat have been linked to damage to the endocrine system, abnormalities in the reproductive system, and the development of lymphoma and leukemia. Despite this, atrazine remains permitted in Latin America [10,11]. In contrast, glyphosate is a non-selective herbicide that interferes with the shikimic acid pathway [12]. In acute cases, its toxicity is considered low, however, it is very harmful to fish due to the surfactant present in its formulation. In chronic cases, evidence suggests that it causes birth defects in certain vertebrate species (acceptable daily dose: 0.05 mg/kg), in addition to being very toxic to other aquatic invertebrates [13]. Therefore, it is important to find safer alternatives to the use of agrochemicals in food production.

Species in the plant kingdom live anchored to the soil, and their ecosystem communication is primarily chemical. This interaction, known as allelopathy, influences the development of the local biome through stimulatory or inhibitory mechanisms. Plant compounds that perform allelopathic functions, especially secondary metabolites are called allelochemicals, and can be used as a more sustainable alternative to agrochemicals [14,15].

One way to verify the impacts of allelopathy is by analyzing DNA alterations. Eukaryotic models are particularly attractive for this purpose since its genetic material is organized into several chromosomes. These models allow for the study of clastogenic and aneugenic damage, as well as the evaluation of the mitotic index and cell death. The *Allium cepa* test is widely used due to its low cost, high sensitivity, and favorable karyotype, characterized by few chromosomes with many base pairs (2*n* = 16), allowing the observation of results with 400× magnification, which is possible to achieve with optical light microscopes [16]. The use of plants as test organisms is validated by the World Health Organization (WHO) and the International Program on Chemical Safety (IPCS) [17].

The Smilacaceae family is mainly composed of angiosperms and comprises more than 700 plant species found throughout the world. The *Smilax* genus is the most numerous in the family, with species popularly known as japicanga and salsaparrilha [18,19,20] widely dispersed in the Brazilian Cerrado. Plants from the Smilacaceae family are used in folk medicine to treat toothache, gonorrhea, leprosy, rheumatism, and syphilis. In addition, they have depurative, diuretic, emollient, expectorant, myotonic, and sudorific activities [20,21,22,23,24]. The main secondary metabolites identified were steroidal saponins, phytosterols, fatty acids, flavonoids, tannins, and alkaloids [20,24,25]. Native to Brazil, *Smilax fluminensis* is used in folk medicine to treat wounds, purify the blood, as an antipyretic, and against syphilis [26,27]. Studies have shown that extracts of this species exhibit antioxidant activity [28], antitumor effects in in vitro and in vivo models [29], and the ability to reverse liver damage caused by paracetamol in mice [30].

Although the genus *Smilax* is well-studied, the biological activities of *S. fluminensis* remain poorly understood. Furthermore, no published studies have evaluated its allelopathic activity. Therefore, this study aimed to characterize and assess the allelopathic, cytogenotoxic, and antigenotoxic activities of the *S. fluminensis* leaf extract and fractions.

## 2. Results and Discussion

### 2.1. Analysis of ^1^H NMR Spectra of S. fluminensis Samples

**Figure 1:**
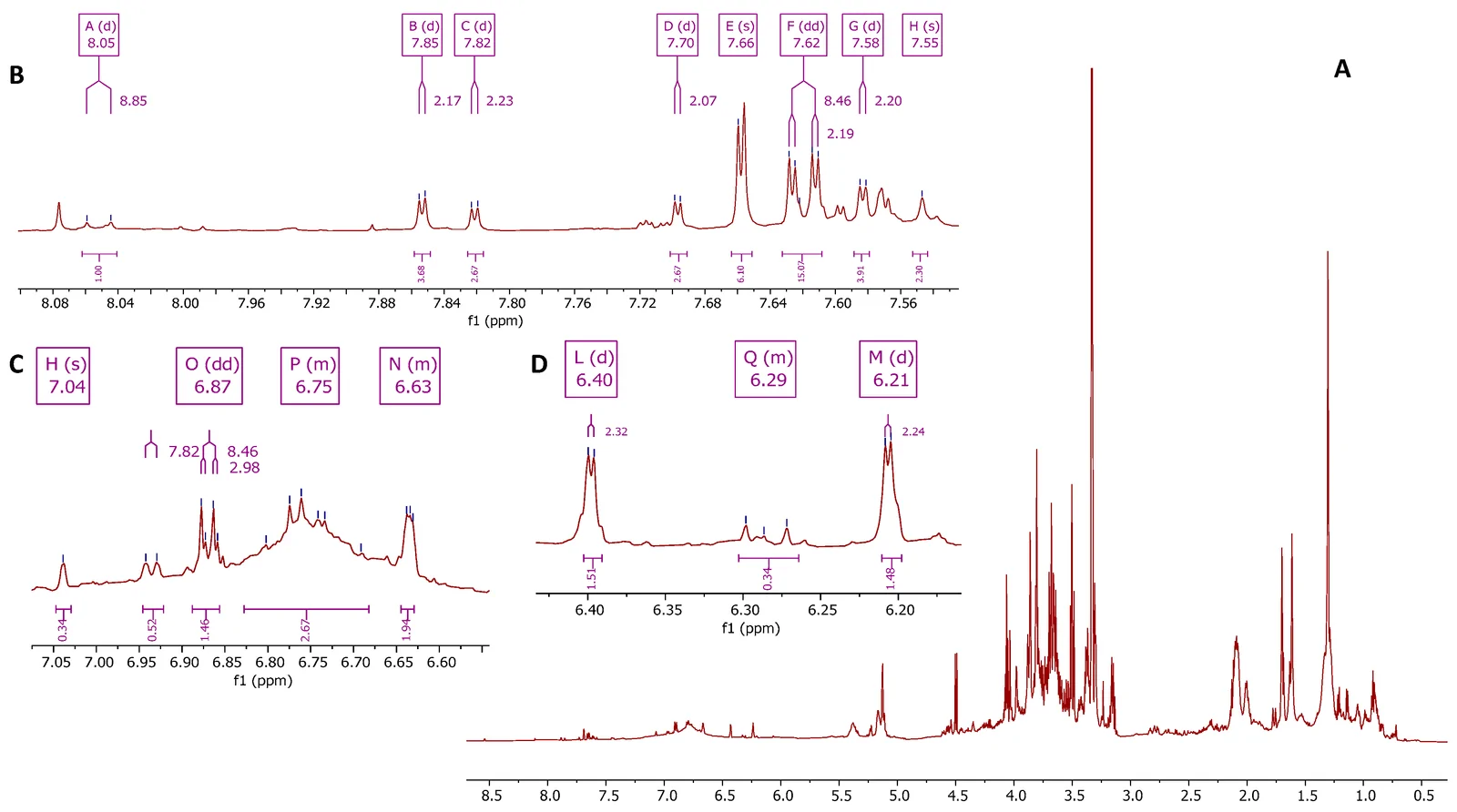
(**A**) ^1^H NMR spectrum of the ethanol extract (EE) of *S. fluminensis* (600 MHz, methanol-*d*_4_ containing 0.01% (*w*/*v*) TSP-*d*_4_); (**B**) expansion between 7.54 and 8.10 ppm; (**C**) expansion between 6.50 and 7.10 ppm, and (**D**) expansion between 6.15 and 6.40 ppm.

The ^1^H NMR spectrum of the ethanol extract (EE) of *S. fluminensis* leaves (Figure 1) showed a clustering of multiple signals with varying intensities between *δ* 0.85 and 8.50, indicating the presence of diverse compounds and multiple functional groups. Most signals appeared in the region typical of carbinolic hydrogens of alcohols (*δ* 3.0–4.2), a characteristic profile of the presence of hydroxylated compounds, such as carbohydrates. Signals in the *δ* 4.5–5.5 region, characteristic of anomeric hydrogens, confirm the presence of carbohydrates and indicate the occurrence of heterosides [31]. Intense signals were also observed in the region of methyl groups characteristic of terpenes and fatty acids (*δ* 0–1.6), which are associated with the hydrogen signals characteristic of carbohydrates, also suggesting the presence of triterpenoid or steroidal saponins. The aromatic region showed typical signals of the presence of flavonoids, such as the signals at *δ* 6.21 (*J* = 2.32 Hz) and *δ* 6.40 (*J* = 2.24 Hz), characteristic of hydrogens H-6 and H-8 of ring A of flavonoids and derivatives, and the signals between *δ* 6.64–8.01, characteristic of ring B of genins, such as quercetin and kaempferol, and of hydrogens of phenylpropanoids. Quercetin in glycosylated form was identified with the following signals: *δ* 7.66 (*d*, *J* = 2.02), 7.62 (*dd*, *J* = 8.46, 2.19 Hz), 6.94 (*d*, *J* = 7.82 Hz), 6.40 (*d*, *J* = 2.32 Hz), and 6.21 (*d*, *J* = 2.24 Hz). Kaempferol derivatives were identified with the signals *δ* 8.05 (*d*, *J* = 8.85 Hz) and 6.87 (*d*, *J* = 8.46 Hz) [32,33] (Figure 1). The LC-DAD-MS analysis of the EE revealed the presence of quercetin, luteolin, and kaempferol derivatives, and saponins, such as steroidal saponin and pseudoprotodioscin or taccaoside A [28,34].

**Figure 2:**
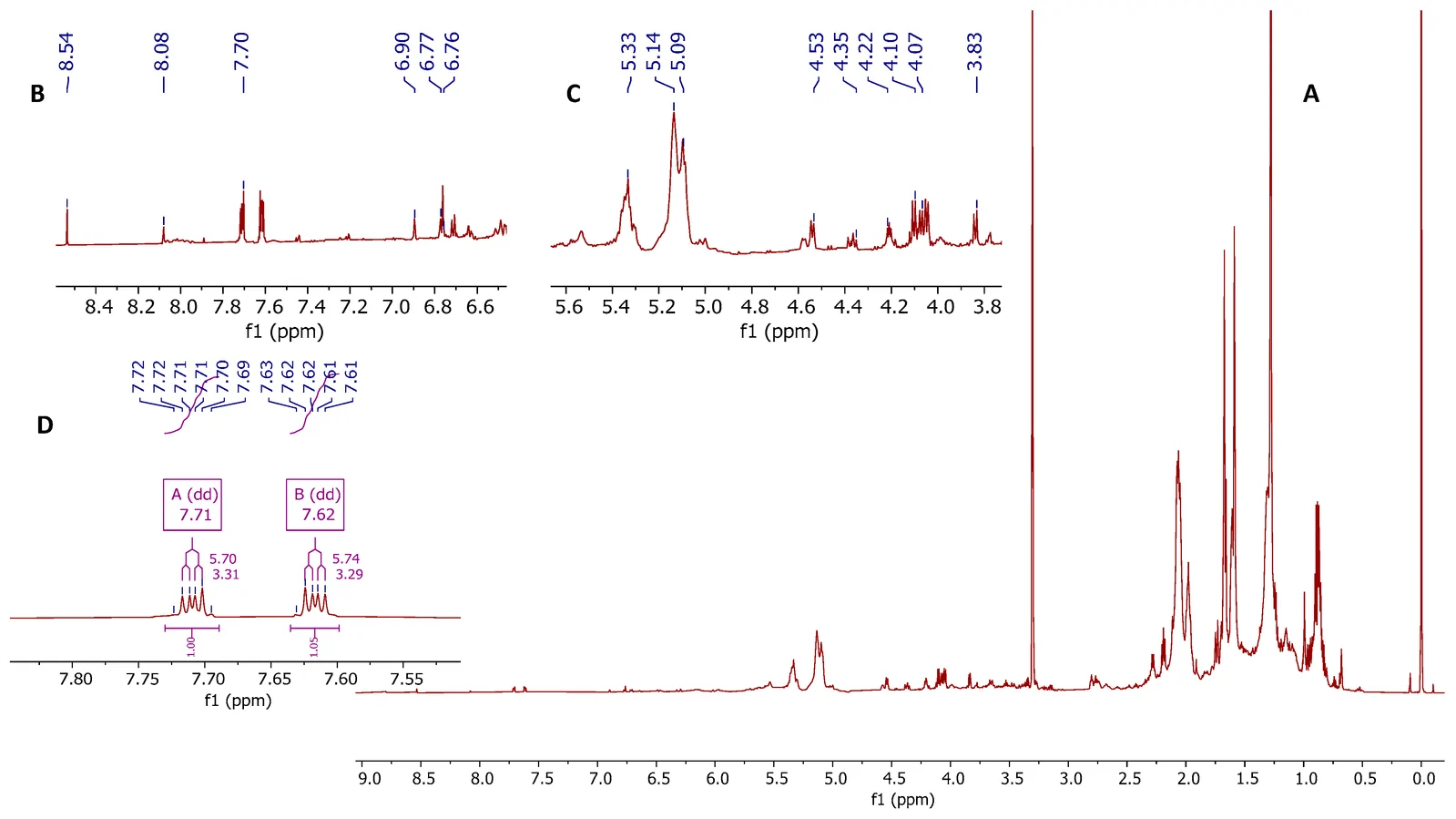
(**A**) ^1^H NMR spectrum of the hexane fraction (HEXF) of *S. fluminensis* (600 MHz, methanol-*d*_4_ containing 0.01% (*w*/*v*) TSP-*d*_4_); (**B**) expansion between 6.5 and 8.6 ppm; (**C**) expansion between 3.8 and 5.6 ppm, and (**D**) expansion between 7.5 and 7.8 ppm.

**Figure 3:**
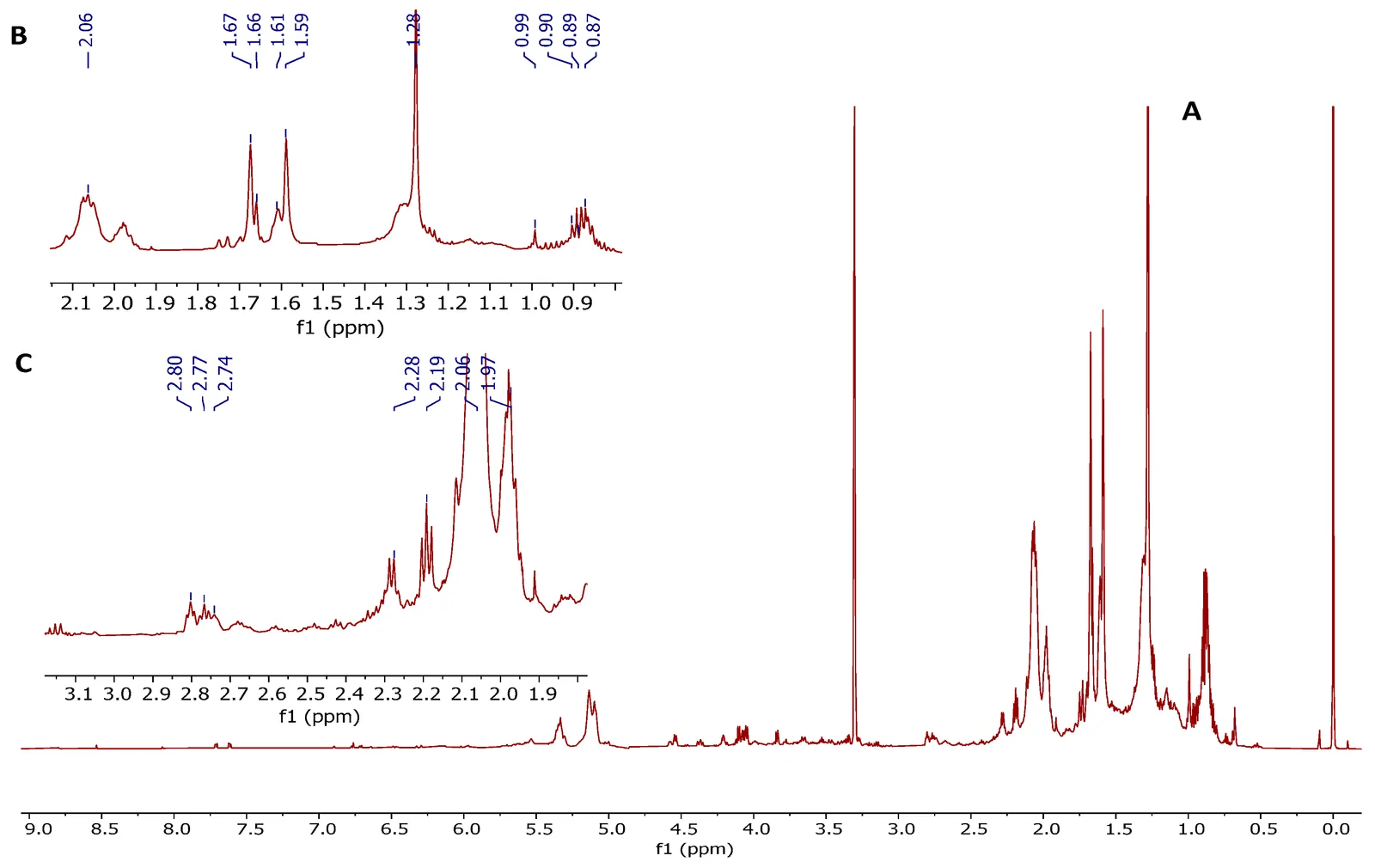
(**A**) ^1^H NMR spectrum of the hexane fraction (HEXF) of *S. fluminensis* (600 MHz, methanol-*d*_4_ containing 0.01% (*w*/*v*) TSP-*d*_4_); (**B**) expansion between 0.8 and 2.1 ppm, and (**C**) expansion between 1.9 and 3.2 ppm.

As expected from EE origin, the hexane fraction (HEXF) primarily showed signals characteristic of aliphatic compounds such as fatty acids (*δ* 0–1.6). However, signals corresponding to aromatic compounds (*δ* 6–9) were also observed (Figure 2B,D), as well as signals that can be attributed to the presence of organic acids or terpenoids (Figure 3B,C). The GC-MS analysis of the HEXF suggested the presence of ethyl esters, such as ethyl linolenate, ethyl palmitate, and ethyl linoleate, and terpenes (phytol) [28,34].

**Figure 4:**
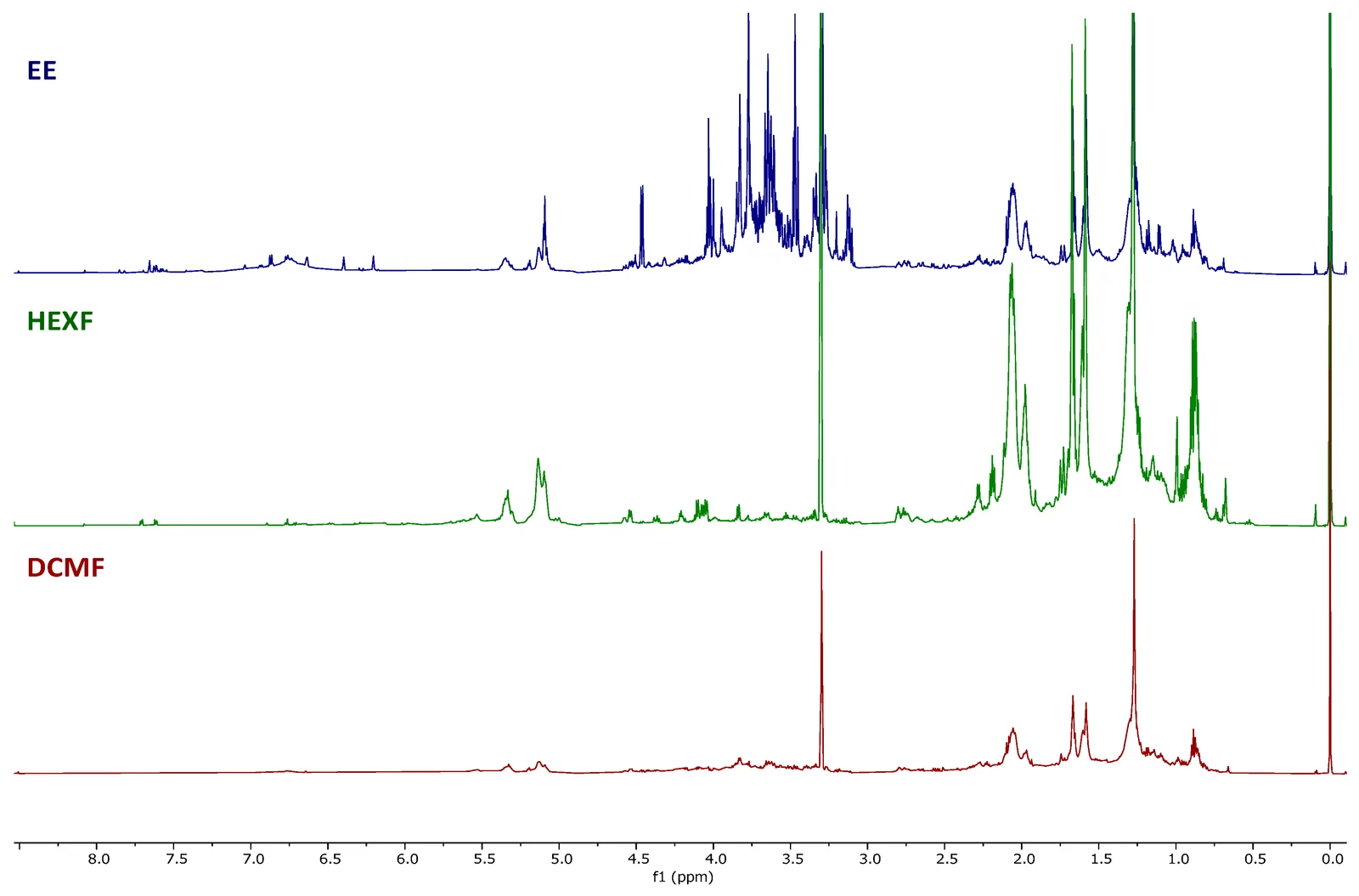
Stacked plot of ^1^H NMR spectra of the ethanol extract (EE), hexane fraction (HEXF), and dichloromethane fraction (DCMF) of *S. fluminensis* (600 MHz, methanol-*d*_4_ containing 0.01% (*w*/*v*) TSP-*d*_4_).

Figure 4 compares two fractions with their original extract. The dichloromethane fraction (DCMF) presented a chemical profile as HEXF, except for the aromatic compounds, presented only in HEXF. Saponins (such as dioscin) have been characterized by LC-DAD-MS as the major compounds in DCMF [34].

**Figure 5:**
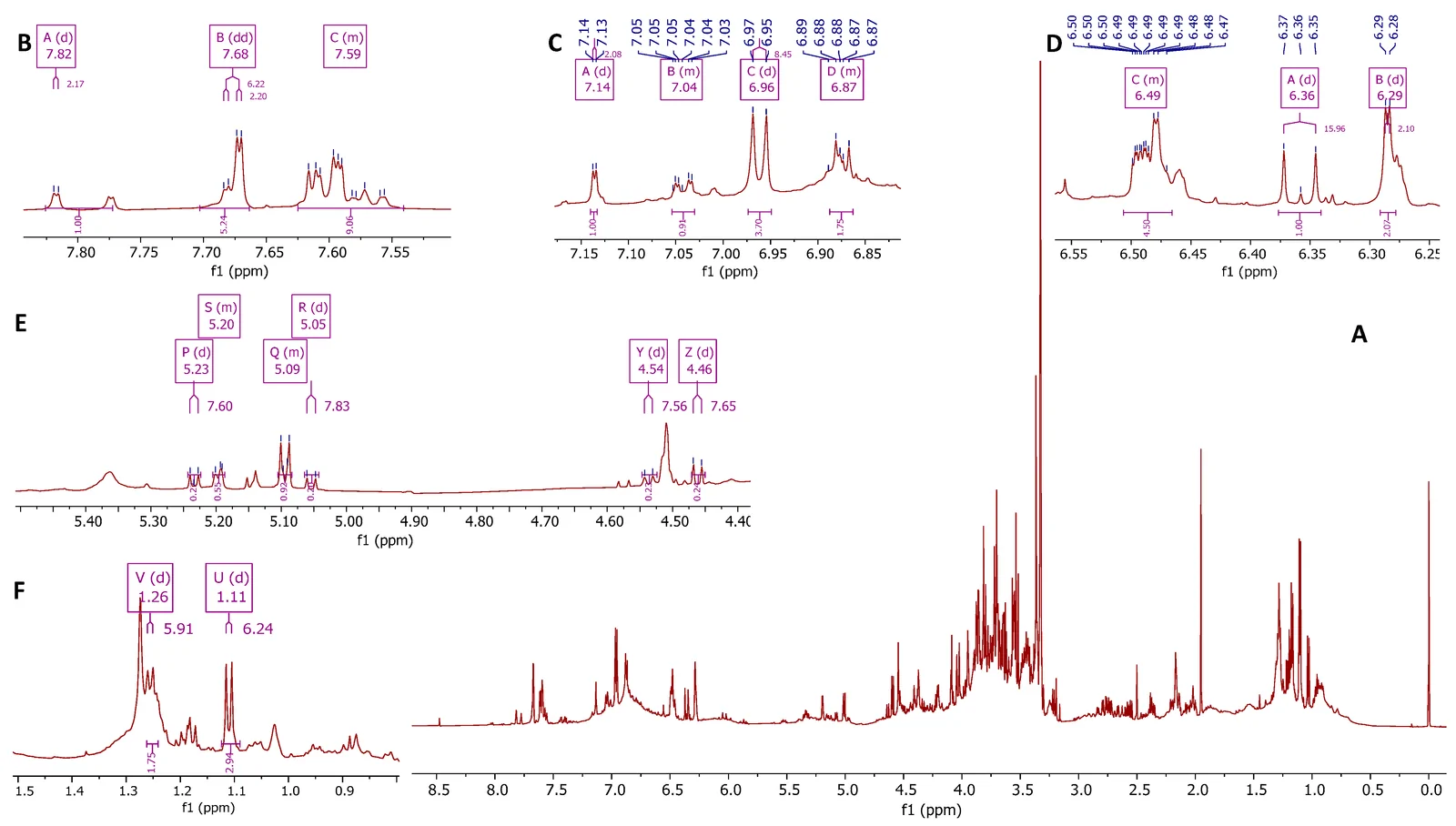
(**A**) ^1^H NMR spectrum of the ethyl acetate fraction (EAF) of *S. fluminensis* (600 MHz, methanol-*d*_4_ containing 0.01% (*w*/*v*) TSP-*d*_4_); (**B**) expansion between 7.5 and 7.9 ppm; (**C**) expansion between 6.85 and 7.15 ppm; (**D**) expansion between 6.25 and 6.55 ppm; (**E**) expansion between 4.40 and 5.50 ppm, and (**F**) expansion between 0.85 and 1.50 ppm.

The ethyl acetate fraction (EAF) showed signals related to aromatic compounds, such as flavonoids, as in the ethanol extract. Figure 5A shows the ^1^H NMR spectrum of the EAF, highlighting the signals in the aromatic region. Figure 5 details the aromatic region (B, C, and D) and the characteristic hydrogens of carbohydrates (E). Most of the signals of the anomeric hydrogens exhibited a coupling constant around 7 Hz, consistent with the *β* configuration of the anomeric hydroxyl group, such as, for example, *β*-glucose (*δ* 4.46; *J* = 7.65 Hz) and *β*-rhamnose (*δ* 5.05; *J* = 7.83 Hz). A doublet at *δ* 1,11 (3H, *J* = 6.24 Hz) (Figure 5F) is characteristic of the methyl group of rhamnose. Comparing the spectra revealed that the EAF is enriched in flavonoids and their glycosides (already described for the ethanol extract). Derivatives of quercetin, luteolin, and kaempferol were the predominant compounds in this fraction by the LC-DAD-MS analysis [28,34].

**Figure 6:**
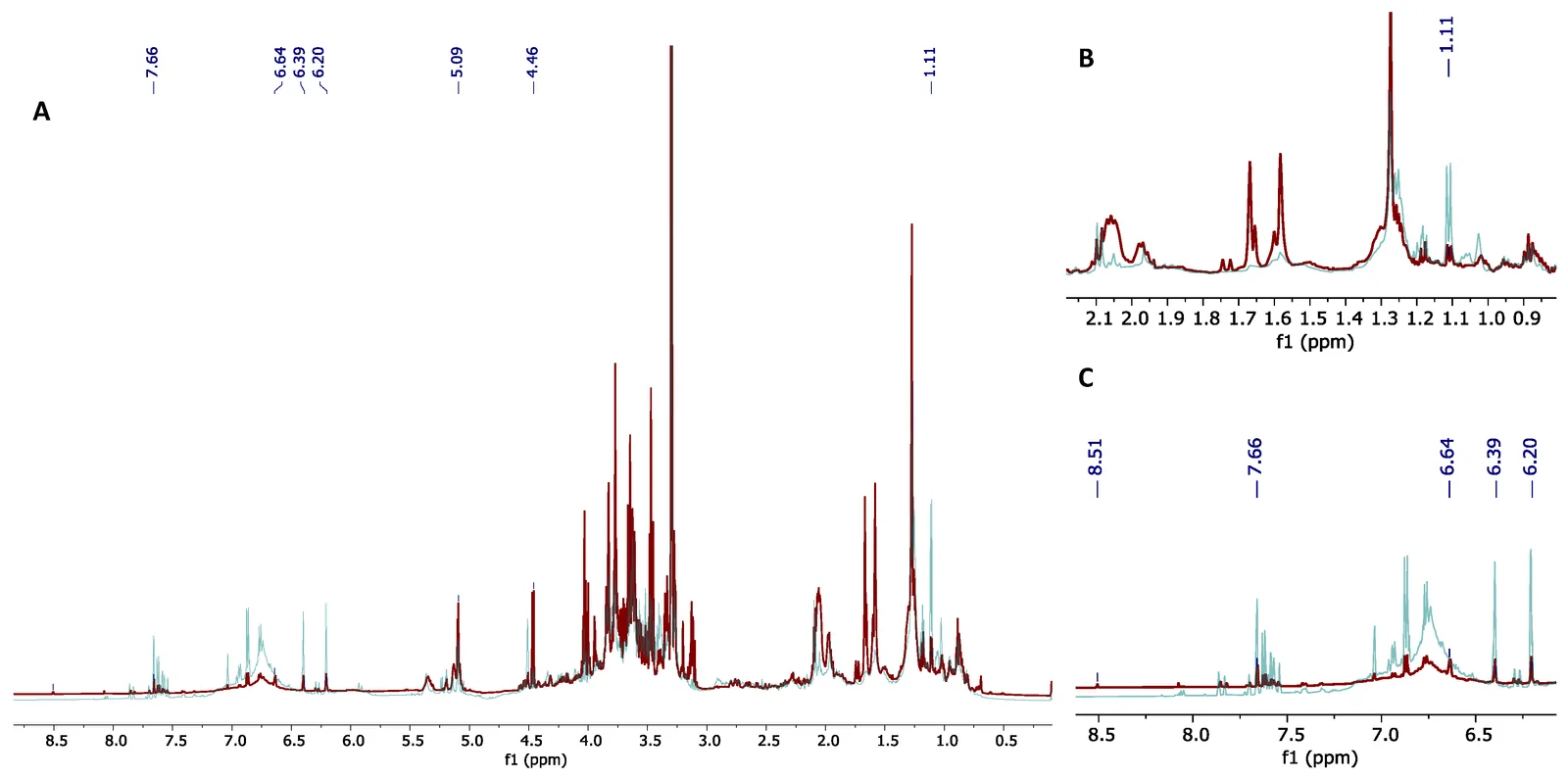
(**A**) ^1^H NMR spectra of the *S. fluminensis* (600 MHz, methanol-*d*_4_ containing 0.01% (*w*/*v*) TSP-*d*_4_) EAF (green) overlaying the EE (red); (**B**) expansion between 0.85 and 2.15 ppm, and (**C**) expansion between 6.0 and 8.5 ppm.

Figure 6 shows the comparison between the EAF spectra and the original EE. The solvent partition enhanced the aromatic compounds in the EAF, especially flavonoids.

**Figure 7:**
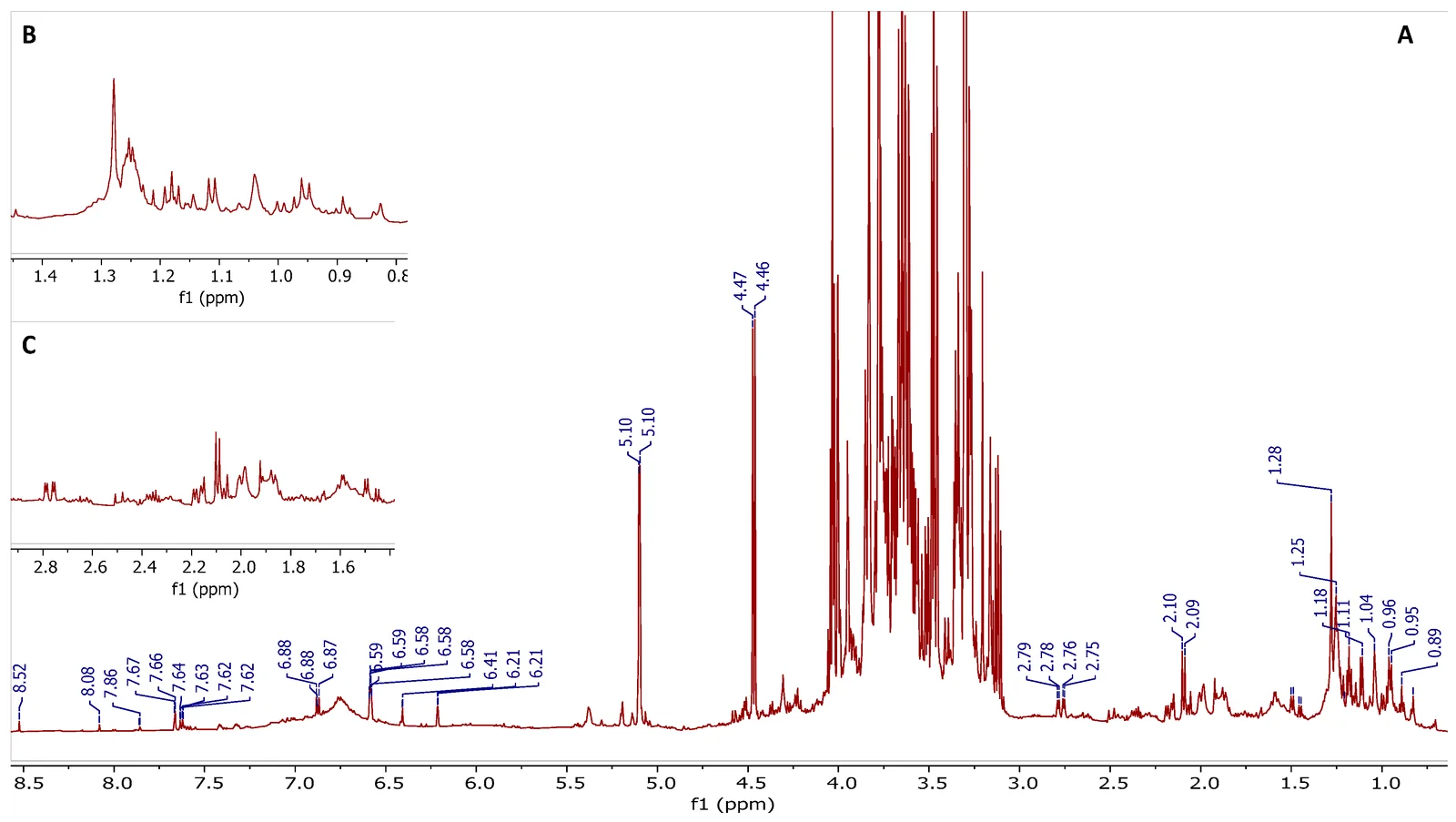
(**A**) ^1^H NMR spectrum of the hydroethanol fraction (HEF) of *S. fluminensis* (600 MHz, methanol-*d*_4_ containing 0.01% (*w*/*v*) TSP-*d*_4_); (**B**) expansion between 0.8 and 1.4 ppm; and (**C**) expansion between 1.5 and 2.9 ppm.

Hydroethanol fraction (HEF) proved to be rich in carbohydrates, with glucose being present in the greatest quantity and identified by the signals of anomeric hydrogens at *δ* 5.10 (*J* = 3.69 Hz, anomer *α*) and *δ* 4.47 (*J* = 7.85 Hz, anomer *β*) (Figure 7). Flavonoids were observed, but in a smaller proportion than in EAF. Figure 7A shows the characteristic signals for these compounds in the region *δ* 6.1–8.6, especially for flavonoids derived from quercetin and kaempferol (already described for the ethanol extract and EAF). The presence of several signals in the aliphatic region *δ* 0.8–2.9, characteristic of methyl groups of steroidal or terpenoid nuclei (Figure 7B,C) and associated with the intense hydrogen signals characteristic of carbohydrates, also suggests the presence of triterpenoid or steroidal saponins in the HEF of *S. fluminensis*. Compounds derived from quercetin, luteolin, kaempferol, steroidal saponin, and pseudoprotodioscin or taccaoside A were predominantly characterized in this fraction [28,34].

### 2.2. Germination Response of Seeds to Extract and Fractions of S. fluminensis

The germination results of *A. cepa* seeds treated with *S. fluminensis* EE and fractions are presented in Table 1. No dose-dependent effects were observed for the first count (vigor), germination rate, or germination speed index (GSI).

Both the EE and fractions of *S. fluminensis* reduced the vigor of *A. cepa* seeds (Table 1). Among the samples tested at 1000 µg/mL, the DCMF was the most effective in reducing vigor (first count), followed by the EAF and HEXF. Compared to the negative control (NC), there was markedly reduced vigor, particularly at 1000 and 750 µg/mL, whereas atrazine (ATZ) increased vigor across all tested concentrations. Statistically, the DCMF stood out, exhibiting significant differences compared to the NC, ATZ, and GLY (*p* < 0.05). Similarly, the EAF and HEXF showed significant differences from the NC and ATZ at all concentrations (*p* < 0.05).

When analyzing the germination rates, the EAF and DCMF (250 and 1000 µg/mL) showed a significant reduction in germination percentage compared to the NC (*p* < 0.05). The EE, HEXF and HEF did not differ significantly from the NC. All samples showed significant differences compared to GLY at the highest concentrations (500, 750, and 1000 µg/mL) (*p* < 0.05), but not in relation to ATZ.

Compared to the NC, the GSI values of all fractions showed significant differences at all concentrations, and EE at 500, 750, and 1000 µg/mL (*p* < 0.05). Differences between GLY and fractions were observed only at higher concentrations (500, 750, and 1000 µg/mL). Additionally, ATZ showed a significant difference in GSI values compared to the NC.

In summary, the *S. fluminensis* samples and GLY decreased the vigor, germination rate, and GSI of *A. cepa* seeds, whereas ATZ only reduced the GSI. Using the same methodology, Amado et al. [35] reported similar results for *Smilax brasiliensis.* For vigor, significant differences were observed in NC for the methanol extract (500 µg/mL), EAF (250 and 500 µg/mL), and hydromethanol fraction (HMF—500 and 750 µg/mL). Furthermore, the methanol extract (250 and 1000 µg/mL) and DCMF (1000 µg/mL) differed significantly from both the NC and GLY, while the HMF (1000 µg/mL) differed significantly from GLY. The GSI results demonstrated significant differences compared to the NC for the methanol extract and EAF (250 and 500 µg/mL). Additionally, the DCMF (1000 µg/mL) showed significant differences compared to the NC and GLY, while the methanol extract, HMF (1000 µg/mL), and DCMF (500 µg/mL) differed significantly from GLY.

The same parameters were evaluated for *L. sativa* seeds (Table 1). GLY was used as a positive control; however, the seeds did not germinate, which prevented us from obtaining results. Similar to the tests performed with *A. cepa*, the first count (vigor), germination rate, and GSI values for *L. sativa* were not dose dependent. Compared to the NC, the EE (500 µg/mL) and DCMF (500 and 1000 µg/mL) showed significant differences in first count values (*p* < 0.05). Regarding ATZ, significant differences were observed for the HEXF (750 and 1000 µg/mL), DCMF (1000 µg/mL), EAF (125 µg/mL), and HEF (125, 500, 750, and 1000 µg/mL) (*p* < 0.05).

Compared to the NC, the germination rates of ATZ showed statistical significance (*p* < 0.05) at all concentrations, followed by EE and DCMF (500 and 1000 µg/mL), HEXF (500 µg/mL), and EAF (1000 µg/mL). HEXF (750 µg/mL), DCMF (1000 µg/mL), EAF (500 µg/mL), and HEF (125, 250, and 500 µg/mL) differed significantly from the ATZ control (*p* < 0.05).

Comparison of the GSI values with NC demonstrated that the EE and DCMF differed significantly (*p* < 0.05) at all tested concentrations, followed by the HEXF (125, 250, and 500 µg/mL), EAF (500, 750, and 1000 µg/mL), and ATZ (125, 500, and 750 µg/mL). Compared to ATZ, DCMF showed the most pronounced differences (500, 750, and 1000 µg/mL), followed by EE (500 and 1000 µg/mL) and the HEF (500 and 750 µg/mL) (*p* < 0.05).

In summary, the *S. fluminensis* samples promoted little change in the vigor of *L. sativa* seeds (limited to the EE and DCMF) but strongly affected the GSI (except for the HEF). The effects of the methanol extract and fractions from *S. brasiliensis* on *L. sativa* seeds were also evaluated by Amado et al. [35]. Consistent with the findings of the present study, a significant difference in seed vigor was observed only for the dichloromethane fraction (1000 µg/mL). In addition, significant differences in the GSI were observed for the methanol extract (750 µg/mL), DCMF (1000 µg/mL), and HMF (500 µg/mL) compared to GLY. Similarly to this study, the main compounds noted in the *S. brasiliensis* samples were phenolic acids and flavonoids derived from quercetin and luteolin [35].

Amado et al. [35] also tested the flavonoid standards quercetin and rutin individually on *L. sativa* and *A. cepa* seeds. Their results demonstrated that these flavonoids did not alter the vigor of *L. sativa* seeds, but significantly affected the first count, germination percentage, and GSI of *A. cepa* seeds, indicating that these substances can delay seed germination. The results of our study are consistent with the data reported by Amado et al. [35]. The findings obtained for the first count, germination rate, and GSI of *A. cepa* and *L. sativa* treated with the extract and fractions of *S. fluminensis* leaves can be explained, at least in part, by the presence of flavonoids in these samples [28,34], although these compounds were not tested in isolation. This finding corroborates the data reported in the literature [35].

### 2.3. Seedling Growth Analysis

**Figure 8:**
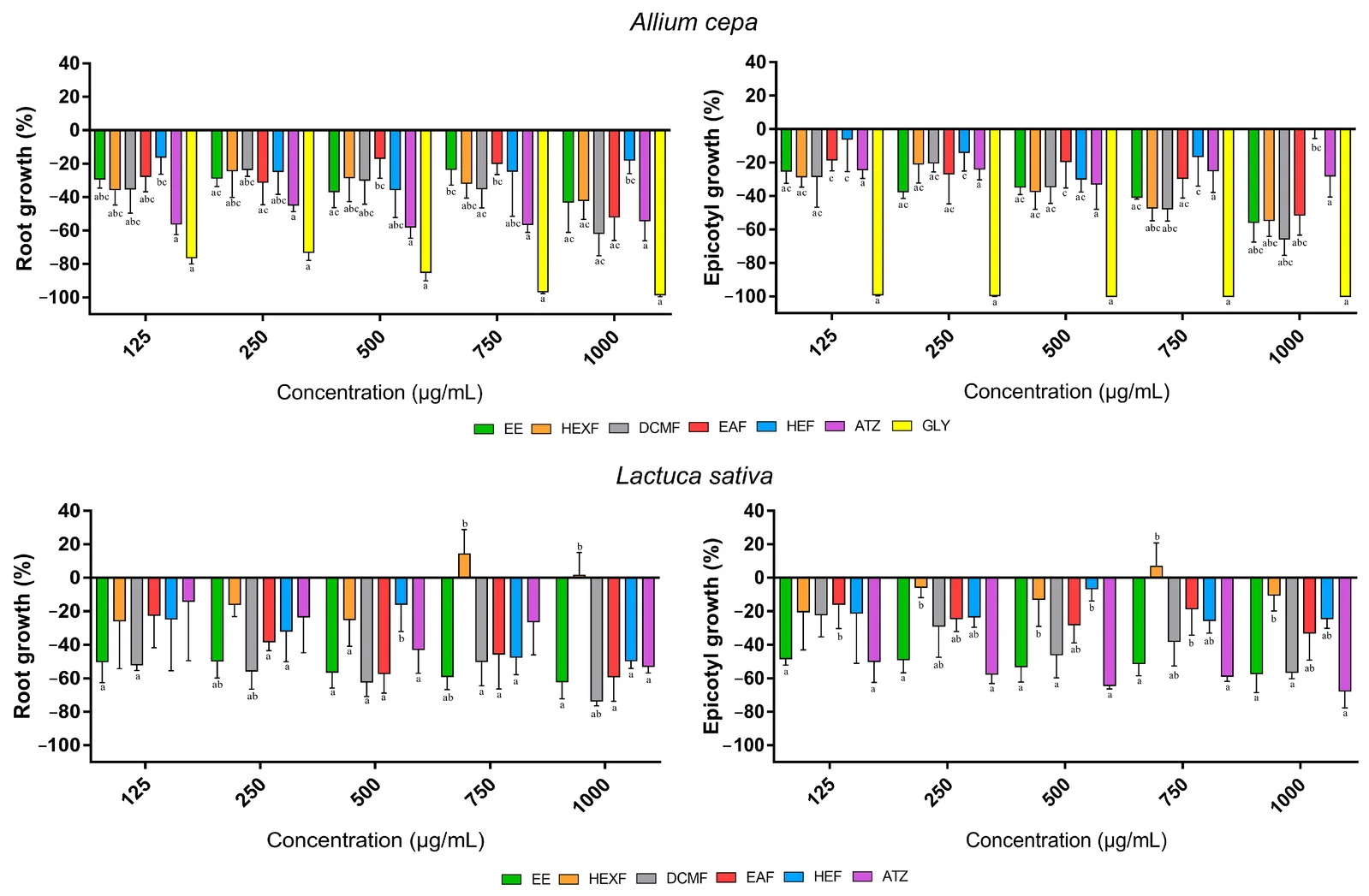
Growth percentage of *Allium cepa* (onion) and *Lactuca sativa* (lettuce) roots and epicotyls treated with *Smilax fluminensis* leaf samples at concentrations of 125, 250, 500, 750, and 1000 μg/mL. EE: ethanol extract, HEXF: hexane fraction, DCMF: dichloromethane fraction, EAF: ethyl acetate fraction, HEF: hydroethanol fraction, ATZ: atrazine, GLY: glyphosate. Results are expressed as mean ± standard error (*n* = 4). ^a^ Statistically significant difference compared to the negative control (*p* < 0.05). ^b^ Statistically significant difference compared to ATZ at the same concentration (*p* < 0.05). ^c^ Statistically significant difference compared to GLY at the same concentration (*p* < 0.05).

The results regarding the epicotyl and root growth of *A. cepa* treated with EE and fractions of *S. fluminensis* leaves are shown in Figure 8. *Smilax fluminensis* samples inhibited the epicotyl and root growth of *A. cepa* at the tested concentrations, except for the HEF (1000 µg/mL) for the epicotyl. Among the samples, the DCMF at 1000 µg/mL promoted the most pronounced reduction in both epicotyl and root development.

Glyphosate was the most effective treatment in inhibiting growth, achieving values close to 100% for the epicotyl and the root. Atrazine was also highly effective in reducing root growth, showing higher inhibition percentages than those observed for the plant extract and fractions.

Compared to the MES, all samples inhibited the growth of *A. cepa* roots (*p* < 0.05), except the EE (750 µg/mL), EAF (500 and 750 µg/mL), and HEF (1000 µg/mL). Similarly, the EE, HEXF, and DCMF reduced epicotyl growth at all concentrations, followed by the EAF (250, 750, and 1000 µg/mL) and HEF (500 µg/mL) (*p* < 0.05). Furthermore, all samples differed significantly from GLY in both root and epicotyl growth (*p* < 0.05).

A study on the ethanol extract and fractions of *S. brasiliensis* leaves obtained by percolation demonstrated an inhibition of *A. cepa* epicotyl and root growth for all samples, with the HEXF being the most active on the epicotyl and the DCMF on the root [36]. Due to their inhibitory activity, the HEXF and DCMF were subsequently fractionated and re-evaluated. The resulting HEXF subfractions from *S. brasiliensis* leaves showed predominantly inhibitory activity on the root and varied effects on the epicotyl of *A. cepa* [37]. Similarly, the DCMF subfractions exhibited a predominant inhibition of epicotyl growth. The main compounds identified in these DCMF subfractions were the fatty acid esters: methyl laurate, methyl 12-methyltridecanoate, methyl palmitate, methyl oleate, and methyl cyclopentanoundecanoate [38].

Amado et al. [39] also evaluated the allelopathic activity of *S. brasiliensis* leaf extracts obtained by Soxhlet extraction. Inhibitory activity on both the epicotyl and root of *A. cepa* was observed when seeds were treated with the methanol extract, ethereal extract, and their respective fatty acids and methyl esters. The compounds identified in these samples were predominantly fatty acid esters. The activity of the methanol extract and fractions from *S. brasiliensis* leaves obtained by Soxhlet was assessed by Amado et al. [35]. The inhibition of *A. cepa* root and epicotyl growth was observed for all tested samples, except for the HEF on the root [35].

*Smilax fluminensis* samples were also evaluated on an eudicotyledonous species (Figure 8). Because *L. sativa* seeds did not germinate in the presence of GLY, the allelopathic activity for this control could not be determined. Regarding the EE and fractions, the *S. fluminensis* samples inhibited both epicotyl and root growth in *L. sativa* at all of the tested concentrations, except for the HEXF (at 750 and 1000 µg/mL for root and 750 µg/mL for epicotyl). Generally, the samples exhibited a higher percentage of root growth inhibition compared to ATZ. In addition, the EE (250 and 750 μg/mL) and DCMF (250 and 1000 μg/mL) were more active than ATZ in inhibiting root growth (*p* < 0.05). Protodioscin inhibited primary root elongation and increased lateral root density in *Arabidopsis thaliana* (an eudicotyledon). This inhibitory effect was associated with the local accumulation of auxin and induced oxidative damage driven by hydrogen peroxide production [40]. Dioscin was the major compound in the DCMF (10.6%) and was also detected in the EAF (6.6%), whereas pseudoprotodioscin or taccaoside A were identified in the EE (12.1%) and EAF (10.0%) [34]. These compounds are steroidal saponins structurally like protodioscin. Although not tested in isolation, these compounds may be partially responsible for the inhibition exhibited by the *S. fluminensis* samples in *L. sativa* and *A. cepa* seeds.

A study with essential oils from *Calotropis procera* branches identified high concentrations of phytol in samples collected in Saudi Arabia (8.73%) and Egypt (38.02%). Negative allelopathic activity was observed on the germination, shoot development, and seedling formation in *Dactyloctenium aegyptium* and *Bidens pilosa*, with the sample from Saudi Arabia being the more active. Therefore, it is possible that the herbicidal activity is not related to phytol [41]. Comparing our results with those of Al-Rowaily et al. [41], it can be inferred that the allelopathic activity of the HEXF from *S. fluminensis* is not due to the phytol present in this fraction, but rather to other compounds, especially ethyl esters [28], which is consistent with the literature data.

Fonseca et al. [36] reported that the ethanol extract and leaf fractions of *S. brasiliensis* obtained by percolation inhibited the epicotyl and root growth of *L. sativa* when treated with the EE and DCMF, while the EAF stimulated both structures. Similarly, for *A. cepa*, they also evaluated the HEXF and DCMF subfractions. The HEXF subfractions demonstrated inhibitory activity on both the epicotyl and root [37], whereas the DCMF subfractions inhibited epicotyl growth and promoted predominant root inhibition [38].

Differently, the methanol and ether extracts and their respective fatty acids and methyl esters obtained by Soxhlet inhibited the *L. sativa* roots growth, with EE being the most active, and stimulated epicotyl growth, with greater activity observed for the methyl esters [39]. The methanol extract and fractions, also obtained via Soxhlet extraction, inhibited root growth across all samples; however, the EAF and HEF stimulated epicotyl growth. The main compounds present in the methanol extract and fractions were phenolic acids and the flavonoid derivatives of quercetin and luteolin [35]. Flavonoids derived from quercetin, luteolin, and kaempferol were characterized in the ethanol extract (EE) and fractions of *S. fluminensis* [28,34], which may explain, at least in part, the allelopathic activity observed for these samples.

### 2.4. Genotoxic Potential of S. fluminensis

The results of the cytotoxic and genotoxic effects for the *S. fluminensis* samples and the controls on *A. cepa* seeds are described in Table 2. ATZ and GLY were used as positive controls in the genotoxicity assay. It is well-documented in the literature that GLY causes genetic damage in meristematic cells [42,43], and that ATZ is correlated with various ecosystem damage [10].

Despite their cost-effectiveness and short assay duration, *A. cepa* tests have limitations. Natural biological variability among individual specimens can affect the reproducibility of results. Due to the plants’ physiological characteristics, extrapolating these findings to humans and other animals requires caution. Furthermore, this model does not allow for the identification of the molecular mechanisms underlying the observed effects, as the results are obtained at the chromosomal level. Additionally, the rapid cell cycle of *A. cepa* makes this test unsuitable for studying the long-term effects [44].

In general, *A. cepa* roots treated with the extract and fractions showed lower mitotic index (MI) values than the negative control and the positive controls at lower concentrations, particularly for the EE (125 and 250 μg/mL) and HEXF (250 μg/mL). Only roots treated with HEF (250 μg/mL) exhibited a higher MI value than GLY (*p* < 0.05). EAF also promoted some higher MI values than the controls, although without a statistically significant difference. Therefore, these findings suggest that the samples negatively interfere with cell division.

Small proportions of chromosomal alterations are commonly observed in nature [45]. Although the chromosomal aberration index (CAI) values for some sample concentrations were higher than those of the negative control, it was more frequent for CAI values to remain lower than those of the positive controls. Roots treated with the EE and fractions exhibited lower CAI values at the lowest concentrations. These results demonstrate that the samples do not exhibit genotoxic activity as they showed no statistically significant differences compared to the negative control. Regarding the necrosis index (NI), the values were, in general, lower than those of the positive controls. Bolle et al. [46] assessed the genotoxic activity of atrazine in *A. cepa*, observing that this agrochemical causes clastogenic and aneugenic damage at concentrations in the ppb range. However, Srivastava and Mishra [47] reported the genotoxic effect of atrazine at concentrations between 15 and 60 μg/mL. The first study on the genotoxic activity of GLY in lettuce was published in 1993; it observed that isolated GLY does not cause genotoxic damage at concentrations between 0.72 and 2.88 μg/mL. However, at the same concentrations, the commercial formulation Roundup is highly genotoxic, indicating that the effect may be driven by the agrochemical’s excipients, such as surfactants [48].

**Figure 9:**
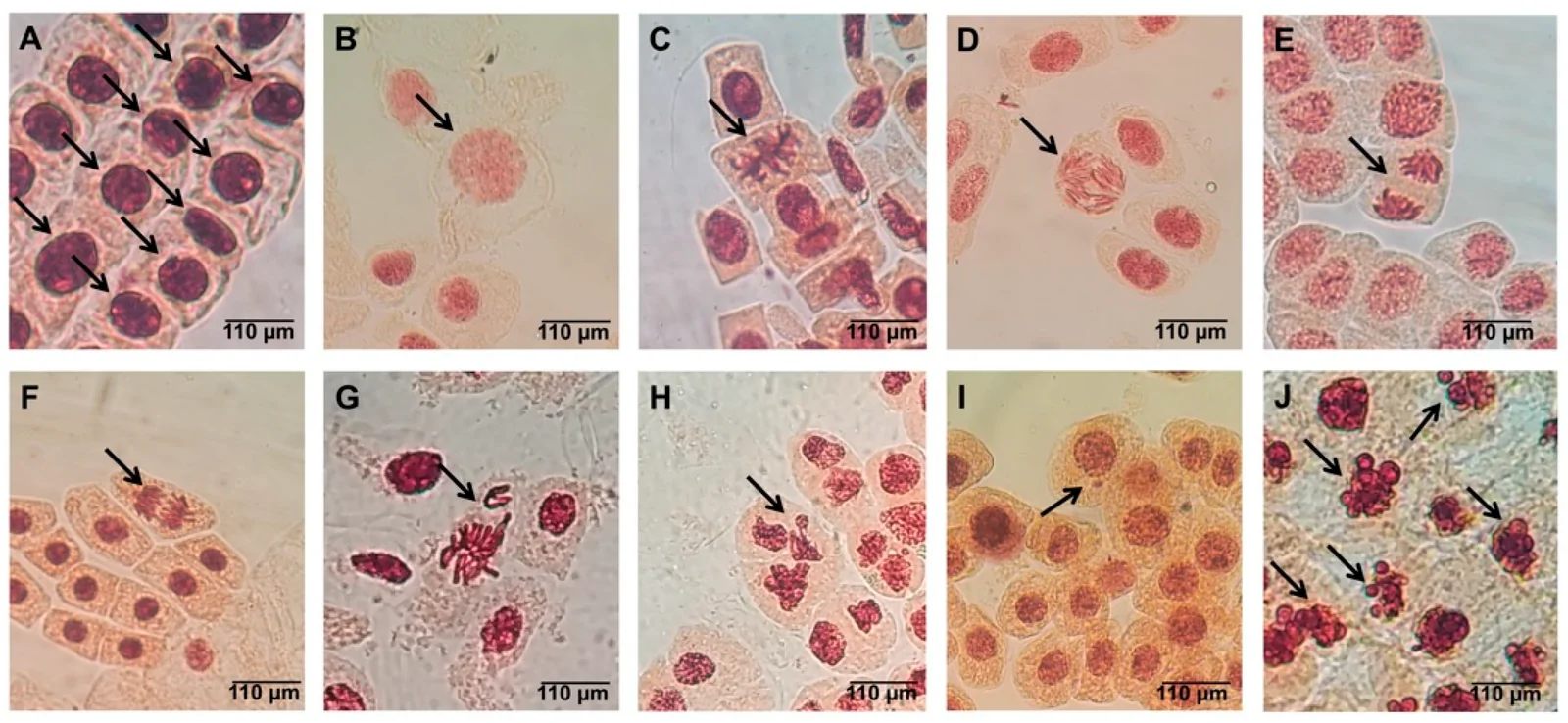
Different cell cycle phases with and without chromosomal abnormalities. (**A**) Normal interphase (HEF 250 μg/mL), (**B**) Normal prophase EE (125 μg/mL), (**C**) Normal metaphase (HEXF 125 μg/mL), (**D**) C-metaphase (EE 125 μg/mL), (**E**) Normal anaphase (DCMF 125 μg/mL), (**F**) Anaphase bridge (DCMF 125 μg/mL), (**G**) Wandering chromosome (GLY 750 μg/mL), (**H**) Aneuploidy (GLY 500 μg/mL), (**I**) Micronuclei (EE 1000 μg/mL), (**J**) Necrosis (GLY 1000 μg/mL). The arrows indicate the chromosomal characteristics found in the cells.

Some chromosomal abnormalities were detected in *A. cepa* roots treated with the *S. fluminensis* leaf extract and fractions (Figure 9). During cell division, microtubules play a critical role in chromosome movement, as they are the protein structures that constitute the cytoskeleton. Chromosomal abnormalities, such as C-metaphase, wandering chromosomes, and chromosome bridges in anaphase (Figure 9D, G, and F, respectively), may be related to a loss of microtubule function. Cell necrosis (Figure 9J) can be observed via light microscopy when the nucleus undergoes irregular and disorganized fragmentation [49]. Additionally, the presence of micronuclei (Figure 9I) indicates aneugenic and/or clastogenic damage [16,50].

Amado et al. [35] evaluated the methanol extract and fractions obtained from *S. brasiliensis* leaves, comparing them with GLY. In contrast to the findings of the present study, their samples stimulated cell division and were identified as genotoxic. Additionally, when the flavonoid standards rutin and quercetin were tested, they increased cell division without demonstrating genotoxicity. Furthermore, the *S. brasiliensis* samples did not cause necrosis [35], which is consistent with the results found in our work.

Another study on *S. brasiliensis* assessed the cytotoxic activity of an ethereal extract and fractions containing fatty acids and methyl esters [39]. A dose-dependent effect was observed for the extract, in which the MI decreased as the concentration increased (evaluating 250, 500, and 1000 μg/mL). Additionally, the methyl esters from the ether extract increased the MI at the highest concentration. Furthermore, *A. cepa* meristematic cells treated individually with commercial standards of methyl linoleate, methyl linolenate, and methyl palmitate did not alter the MI. Therefore, the combination of fatty acid methyl esters demonstrates a synergistic cytotoxic effect in *A. cepa* meristematic cells [39]. In our study, we observed an increase in the MI at the two highest concentrations of the HEXF, which is consistent with the findings of Amado et al. [39], since this fraction is mainly composed of a mixture of ethyl esters.

Studies have demonstrated the antitumoral activity of several plant secondary metabolites that act by preventing cell division. Steroidal saponins extracted from species of the genus *Tacca* (known as taccalonoides) inhibit the deacetylation of histone proteins and act on microtubules, thereby preventing DNA replication [51,52]. Additionally, dioscin has been shown to induce cell death via intracellular iron accumulation [53]. Our findings are consistent with these literature data, as dioscin was present in the DCMF and EAF, whereas pseudoprotodioscin or taccaoside A was characterized in the EE and HEF of *Smilax fluminensis* [28,34], explaining, at least in part, the decrease in MI values in *A. cepa* roots treated with these samples at most of the concentrations tested when compared to the negative control.

### 2.5. Antigenotoxic Potential of S. fluminensis

The results for the antigenotoxicity assay are presented in Table 3. In the antigenotoxicity assay using seeds pre-treated with ATZ, *A. cepa* roots treated with EE and its fractions generally exhibited lower MI values than the negative control and ATZ. Conversely, EE (1000 μg/mL) and DCMF (750 μg/mL) showed higher MI than ATZ. Among the fractions, the HEXF, EAF, and HEF showed a reduction in MI in all concentrations.

In general, lower CAI values were observed for the EE and its fractions compared to the positive control. Specifically, the EE (750 µg/mL), HEXF (750 and 1000 µg/mL), DCMF (1000 µg/mL), and EAF (125, 500, 750, and 1000 µg/mL) significantly reduced the CAI when compared to ATZ (*p* < 0.05). Except for the HEF (500 μg/mL), all samples showed a percentage reduction in CAI, demonstrating an antigenotoxic effect. EAF stood out by exhibiting values above 100% at all tested concentrations, except for 250 μg/mL.

The mechanism of action of ATZ is well-described in the literature. Triazine derivatives act as herbicides by interfering with photosystem II and binding to the D_1_ subunit of plastoquinone, culminating in a reduction in carbon fixation [54].

Furthermore, the EE and polar fractions, particularly the EAF and HEF, exhibited antigenotoxic activity following atrazine pretreatment. This protective effect may be related to the major compounds present in these samples, such as quercetin, luteolin, and kaempferol derivatives, as well as saponins (including dioscin in EAF, pseudoprotodioscin or taccaoside A and steroidal saponin in EE and HEF) [34]. Bioactive compounds found in plant extracts can exhibit antigenotoxic potential by protecting DNA against genotoxic damage. Antioxidant substances can minimize this damage by serving as the primary defense against oxidative stress, maintaining the integrity of the genetic material and preventing genotoxic events [55]. Flavonoids and saponins are well-known for their antioxidant activity; they can donate electrons or scavenge free radicals through direct interaction via hydroxyl groups. This mechanism activates antioxidant enzymes, such as nitric oxide synthase, while inhibiting pro-oxidant ones, including xanthine oxidase and protein kinase C [56]. Furthermore, saponins can chelate free metal ions like copper and iron, which act as enzymatic cofactors for pro-oxidant enzymes [57,58]. In addition, flavonoids inhibit cell proliferation by interfering with signaling pathways involved in cell growth and division. They can also induce cell cycle arrest, typically at the G2/M or G1/S checkpoints, preventing cells from completing their replication cycle [59].

Regarding the antigenotoxicity assay on seeds previously treated with GLY, in general, the EE and fractions promoted higher MI values than the controls. The DCMF stood out by exhibiting higher MI values at the lowest concentrations. The EAF (125 and 500 µg/mL) and HEF (125 µg/mL) significantly reduced the CAI (*p* < 0.05) (Table 3).

All samples showed higher CAI values than the NC at the tested concentrations. Although some concentrations showed higher CAI values than the positive control, they reduced the CAI (antigenotoxic effect), especially at the lower concentrations. According to Verschaere and Van Staden [60], a reduction in CAI values between 25 and 40% indicates a moderate antigenotoxic effect, whereas values above 40% demonstrate a strong effect. To demonstrate the antigenotoxic effect, we considered the CAI values of *S. fluminensis* samples that showed statistically significant differences (*p* < 0.05) compared to the positive controls (ATZ and GLY), which were used to calculate the low CAI (%). Therefore, the results of this study indicate that the following *S. fluminensis* samples exhibited an antigenotoxic effect in the ATZ assay: EE (125 and 750 µg/mL), HEXF (750 and 1000 µg/mL), DCMF (125, 250, and 1000 µg/mL), EAF (at all tested concentrations), and HEF (125, 250, and 750 µg/mL). In the GLY assay, the EAF (125 and 500 µg/mL) and HEF (125 µg/mL) showed an antigenotoxic effect (Table 3). For the remaining samples with low CAI values ≥ 25%, our findings suggest that they may possess antigenotoxic potential.

GLY is a non-selective herbicide that interferes with the shikimic acid pathway by inhibiting the enzyme 5-enolpyruvylshikimate-3-phosphate synthase, preventing its binding to phosphoenolpyruvate, and consequently, preventing or strongly reducing the synthesis of the essential amino acids tryptophan, tyrosine, and phenylalanine [12]. It was observed that none of the samples significantly decreased CAI following treatment with this herbicide.

A study performed by Amado et al. [35] evaluated the antigenotoxic activity in the glyphosate assay using extracts and fractions of *S. brasiliensis* rich in flavonoid derivatives, including *O*-deoxyhexosyl-hexosyl-quercetin, 3-*O*-*β*-galactopyranosyl-quercetin, *O*-deoxyhexosyl-hexosyl-quercetin, 3-*O*-*β*-glucopyranosyl-quercetin, and *O*-deoxyhexosyl-hexosyl-luteolin. These samples demonstrated antigenotoxic effects at concentrations ranging from 250 to 1000 µg/mL. Additionally, this study showed the antigenotoxic effect of commercial flavonoid standards, specifically quercetin and rutin. Our results corroborate the results obtained by Amado et al. [35], given that a prior phytochemical analysis of S. fluminensis characterized quercetin and luteolin (flavonoid derivatives) as major compounds in the extract and polar fractions [34]. Thus, we suggest that the antigenotoxic activity of *S. fluminensis* is attributed, at least in part, to flavonoid derivatives.

**Figure 10:**
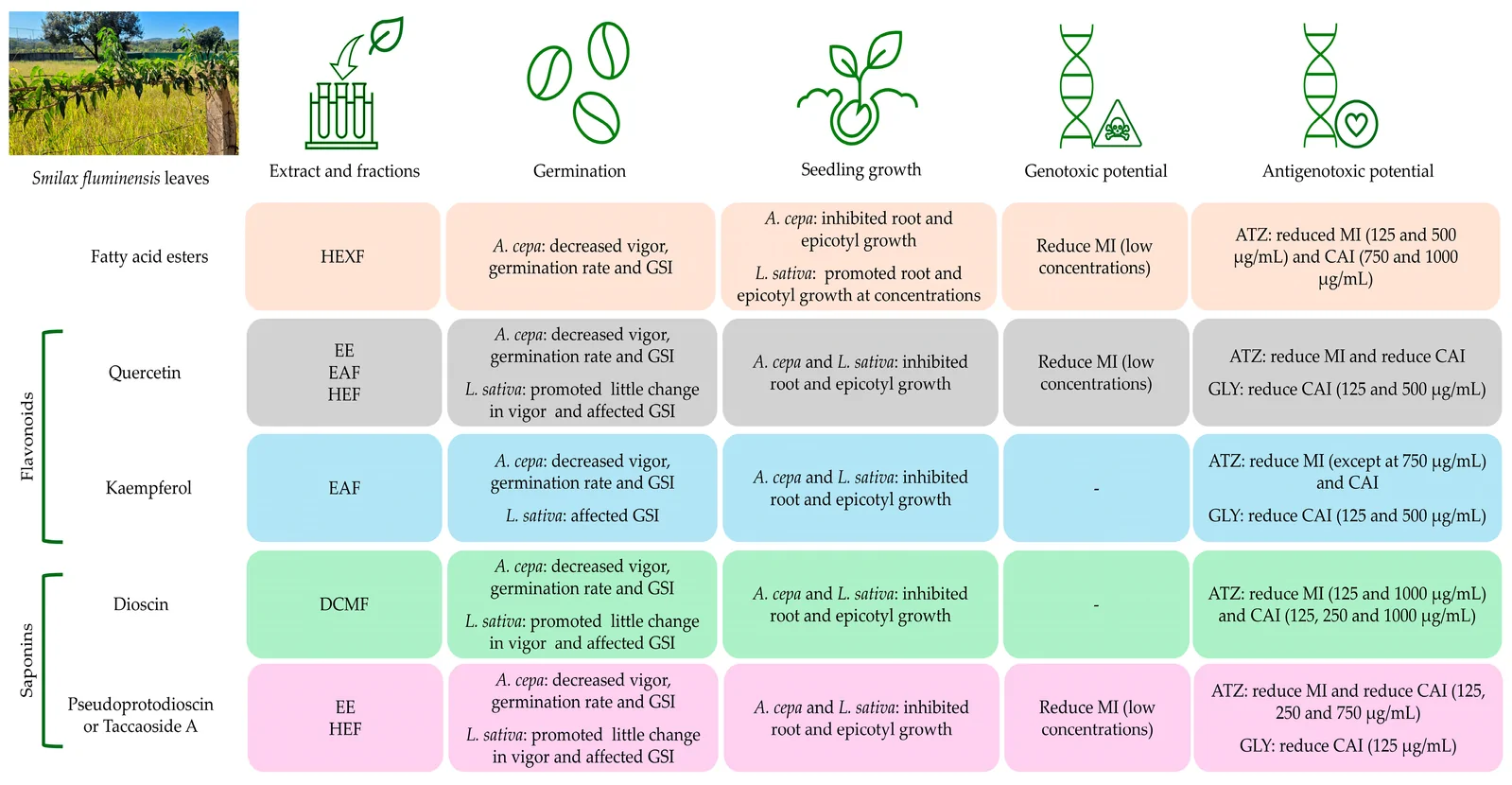
Schematic diagram summarizing the relationship between the characterized phytochemicals and their proposed biological effects. EE: ethanol extract; HEXF: hexane fraction; DCMF: dichloromethane fraction; EAF: ethyl acetate fraction; HEF: hydroethanol fraction; GSI: germination speed index; MI: mitotic index; CAI: chromosomal aberration index; ATZ: atrazine; GLY: glyphosate.

Figure 10 summarizes the results presented in this investigation, highlighting the correlation between the characterized compounds and their proposed biological effects.

## 3. Materials and Methods

### 3.1. Chemicals

Ethanol (C_2_H_6_O) was purchased from Vetec (Duque de Caxias, RJ, Brazil); hexane (C_6_H_14_), dichloromethane (CH_2_Cl_2_), ethyl acetate (C_4_H_8_O_2_), hydrochloric acid (HCl), sodium hydroxide (NaOH), and glacial acetic acid (C_2_H_4_O_2_) were acquired from CRQ—Cromato Produtos Químicos (São Paulo, SP, Brazil). Methanol-*d*_4_, 3-(trimethylsilyl) propionic-2,2,3,3-*d*_4_ acid sodium salt (TSP-*d*_4_), and 2-(*N*-morpholino) ethanessulfonic acid (C_6_H_12_NNaO_4_S) were purchased from Sigma-Aldrich (Darmstadt, Germany). Glyphosate (C_3_H_7_NO_5_P) was obtained from Citromax (São Lourenço do Oeste, SC, Brazil), atrazine (C_8_H_14_ClN_5_) from Nortox (Arapongas, PR, Brazil), and orcein (C_28_H_24_N_2_O_7_) from Himedia (Kelton, PA, USA).

### 3.2. Collection and Preparation of Plant Material

*S. fluminensis* leaves were collected in Ijaci, southern region of Minas Gerais State, Brazil (21°13′46″ S, 44°55′6″ W), in October 2014, according to proof of registration for the collection of botanical material carried out with SISBIO no. 24542-5, on 20 March 2018. Fertile samples were collected, and the vouchers were identified by Regina Helena Posch Andreata at the PAMG Herbarium, at the Agricultural Research Company of Minas Gerais (EPAMIG), as *Smilax fluminensis* Steud. (PAMG 57133). This work has authorization to access the genetic heritage with Access Registration No. A605DF4, dated 13 December 2022, granted by SISGEN/CGEN/MMA, by the Brazilian Biodiversity Law (13.123/2015).

The fresh leaves (444.49 g) were dried in an oven (Edutec FDC-1000 Serials, Sapucaia do Sul, RS, Brazil) at 35 °C for 7 days to stabilize the biomass (114.26 g).

The extract and fractionating processes were carried out according to the methodology of Morais et al. [61]. Initially, 86.5 g of the material was extracted in a percolation with ethanol until depletion. After extraction, the filtrate obtained was placed in a rotary evaporator (IKA HB10 digital, Staufen, Germany) at 40 °C until 14.376 g of dried extract was obtained. Subsequently, 8 g of the ethanol extract (EE) was solubilized in 160 mL of ethanol/water (7:3, *v*/*v*) and partitioned with solvents in increasing order of polarity, using hexane, dichloromethane, and ethyl acetate to obtain the hexane (HEXF, 0.337 g), dichloromethane (DCMF, 1.689 g), ethyl acetate (EAF, 1.170 g) and hydroethanol (HEF, 4.269 g) fractions.

### 3.3. Nuclear Magnetic Resonance (NMR) of the Ethanol Extract and Its Fractions

Samples (15 mg) were dissolved in 800 μL of methanol-*d*_4_ containing 0.01% (*w*/*v*) TSP-*d*_4_ (3-(trimethylsilyl) propionic-2,2,3,3-*d*_4_ acid sodium salt), vortexed (Vortex mixer, Global Trade Technology XH-C, Jaboticabal, SP, Brazil) for 1 min, and placed in an ultrasound bath (Sanders SoniClean, Santa Rita do Sapucaí, MG, Brazil) for 20 min. After centrifugation at 17,000 *g* for 15 min, the supernatants (700 μL) were transferred to 5 mm diameter NMR tubes. The ^1^H NMR experiments were performed in a Bruker Avance Neo 600 MHz (Fällanden, Switzerland), and the spectra were acquired at 300 K with a spectral window (SW) of 16 ppm, a number of digitized points (TD) of 64 K, with HDO (deuterated water) signal pre-saturation, acquisition number (NS) of 128, acquisition (AQ), and waiting times before each acquisition (d1) of 3.2 s and 5.0 s, respectively. All spectra were obtained using the zgcppr pulse sequence and processed using a 0.3 Hz line broadening before the Fourier transform. The phases and baselines were automatically corrected using the TopSpin 4.4.0 program. Finally, the spectra were calibrated using the TSP-*d*_4_ signal at 0.00 ppm.

### 3.4. Analysis of Seed Germination

The tests were carried out with seeds of eudicotyledonous *Lactuca sativa* (lettuce) var. aurelia (Topseed, Lot: 073792, São Paulo, SP, Brazil) and monocotyledonous *Allium cepa* (onion) cv. pear-shaped bay (Topseed, Lot: 073432, São Paulo, SP, Brazil). No sanitizing process was performed on the seeds prior to germination. Germination and growth were conducted in 2-(*N*-morpholino) ethanessulfonic acid (MES) buffer solution with a pH adjusted to 6.0–6.2 with a NaOH 1 M solution. The extract and fractions of *S. fluminensis* and the positive controls were tested at concentrations of 0, 125, 250, 500, 750, and 1000 µg/mL solubilized in MES buffer solution in quadruplicate [35]. Thus, 25 seeds (*n* = 3600 seeds of each species) were placed on disposable and sterile Petri dishes (90 × 15 mm) containing filter paper and 4 mL of buffer solution containing *S. fluminensis* samples or 4 mL of the control (negative control: MES; and positive controls: atrazine (ATZ) and glyphosate (GLY) for *A. cepa* and atrazine for *L. sativa*).

The Petri dishes were closed, sealed with film paper, and incubated in a biochemical oxygen demand (BOD) incubator germination chamber (Cienlab, Campinas, SP, Brazil) in the dark at 25 °C for 12 days for *A. cepa* seeds and 7 days for *L. sativa* seeds [62]. The number of germinated seeds was counted daily. Based on these data, the germination speed index (GSI) was calculated using the following equation [63]:

(1) $$GSI=\frac{G1}{N1}+\frac{G2}{N2}+\cdots \frac{Gn}{Nn}$$

where G1, G2, Gn = number of germinated seeds; N1, N2, Nn = number of days after sowing.

In the “first count” parameter, seeds germinated up to the 4th day for *L. sativa* and up to the 6th day for *A. cepa* were counted, and seed vigor was observed. The number of seeds germinated was used to determine the “germination potential” [64]. Analyses were performed in quadruplicate, and pure MES solution was evaluated as a negative control.

The first count (seed vigor) was calculated using the following equation:

(2) $$First\;count=\frac{total\;number\;of\;seeds’\;germinated\;up\;to\;the\;n\;day}{total\;number\;of\;seeds}\times 100$$

where *n* = 4th for *L. sativa*, and *n* = 6th for *A. cepa*.

The germination rate was calculated using the following equation:

(3) $$Germination\;rate=\frac{total\;number\;of\;seeds\;germinated}{total\;number\;of\;seeds}\times 100$$

### 3.5. Seedling Growth Analysis

Once the total germination time for *L. sativa* and *A. cepa* was completed, the plates were removed from the germination chamber and cooled to −20 °C for 24 h to interrupt the growth process. Then, the length of each root and epicotyl was measured, in millimeters, using a ruler. Subsequently, the effects on the growth of seedlings were calculated using the following formula [65]:

(4) % of growth = [(Ma−Mc)/Mc]×100

where Ma is the average root or epicotyl length of the seedlings and Mc is the average root or epicotyl length of the seedlings for the negative control (MES). Thus, positive values represent stimulation of the studied parameter, and negative values represent inhibition.

### 3.6. Cytogenotoxicity Assay

Initially, 30 *A. cepa* seeds were distributed in each Petri dish containing filter paper and 4 mL of distilled water and allowed to germinate. No sanitizing process was performed on the seeds prior to germination. After 12 days in a BOD incubator, in the dark, at 25 °C, 10 seedlings were transferred to each Petri dish containing 4 mL of the respective extract, fraction, or control solution. Five samples of *S. fluminensis* were evaluated at five concentrations (125, 250, 500, 750, and 1000 μg/mL), with three independent replicates, resulting in 75 Petri dishes. MES buffer was used as the negative control, while glyphosate (GLY) and atrazine (ATZ) were used as positive controls and were also evaluated in triplicate at the same five concentrations. In total, 108 Petri dishes containing 1080 seedlings were used. The seedlings were maintained under the respective experimental conditions until the roots reached approximately 1–2 mm, suitable for the collection of meristematic regions for analysis [35].

After 24 h in the presence of the samples, the roots were collected, fixed in 1 mL of Carnoy reagent (ethanol:glacial acetic acid, 3:1, *v*/*v*) in 2-mL microtubes and stored in a freezer (Consul CRM37EBANA, Joinville, SC, Brazil) at −20 °C until slide preparation.

### 3.7. Antigenotoxicity Assay

The antigenotoxicity assay was adapted from Sousa et al. [66] using a post-treatment exposure protocol. Initially, 30 *A. cepa* seeds were placed to germinate in Petri dishes containing filter paper and 4 mL of distilled water, without prior sanitization. After 12 days in a BOD incubator at 25 °C, 10 seedlings were transferred to Petri dishes containing 4 mL of GLY or ATZ at 500 μg/mL. This concentration was selected based on the high chromosomal aberration index (CAI) and low necrosis index (NI) (observed in the Section 3.8). After 24 h, the seedlings were washed with distilled water and exposed to the samples of *S. fluminensis* at 125, 250, 500, 750, and 1000 μg/mL for an additional 24 h. All treatments were performed in triplicate. The roots were then collected, fixed in 1 mL of Carnoy’s solution, and stored at −20 °C until slide preparation and analysis. A total of 810 seedlings were used for the antigenotoxicity assay for each of the positive controls (GLY or ATZ).

### 3.8. Preparation of Glass Slides

At least 3 root tips of each triplicate were selected to prepare the glass slides (*n* ≥ 108 root tips to cytogenotoxicity assay and *n* ≥ 159 root tips to antigenotoxicity assay) [67]. First, they were washed in distilled water for 5 min, then hydrolyzed with 1 N HCl for 15 min in a water bath (Nova Instruments 1236, Piracicaba, SP, Brazil) at 40 °C. The roots were washed with ice-cold distilled water for 5 min. Subsequently, the root’s apical meristems were transferred to the slide containing 1 drop of 2% orcein previously diluted in acetic acid/water (11:9, *v*/*v*).

The roots were chopped with surgical blades and crushed. Afterward, the slides were analyzed under a Primo Star Zeiss trinocular light optical microscope (Oberkochen, Germany) at 40× magnification coupled to a Canon PowerShot A650 IS camera, 12.1 Megapixels, 6× optical zoom (Hong Kong, China). Cell counting was performed using ImageJ software v.1.54 (National Institutes of Health). In each concentration (125, 250, 500, 750, and 1000 μg/mL) and negative control, 2100 cells (700 cells per replicate) were analyzed using the scanning technique (*n* = 75,600 cells to cytogenotoxicity assay and *n* = 111,300 cells to antigenotoxicity assay).

In the antigenotoxicity test using 500 μg/mL of GLY, the protocol for preparing slides differed due to the sensitivity of the roots to the treatment. Thus, the root tips were washed with distilled water for 5 min, hydrolyzed with 1 N HCl for 3 min in a water bath at 30 °C, and washed again with ice-cold distilled water for 5 min. Subsequently, the previously described crushing and staining techniques were performed.

The parameters analyzed during cell counting were the mitotic index (MI), chromosomal aberrations index (CAI), and necrosis index (NI). The types of chromosomal aberrations evaluated for each phase of the cell division cycle: interphase (presence of heterochromatins, micronuclei, nuclear bonds, and presence of more than three nucleoli), prophase (presence of micronuclei, wandering chromosomes, fragment of chromosomes and aneuploidy), metaphase (sticky chromosomes, micronuclei, wandering chromosomes, C-metaphase, aneuploidies, and polyploidies), anaphase (micronuclei, wandering chromosomes, anaphase bridges and lagging chromosomes), and telophase (micronuclei, wandering chromosomes, aneuploidies, and heterochromatins) [68,69,70]. The samples were considered cytotoxic when the MI was statistically lower when compared to the NC. Conversely, samples were considered genotoxic when the CAI was significantly higher when compared to the NC.

The mitotic index was calculated by the equation [71]:

(5) MI=(m/T)×100

where *m* is the number of cells in mitosis, and *T* is the total number of cells.

The chromosomal aberration index was obtained by the following equation [71]:

(6) CAI=(a/T)×100

where *a* is the number of cells with aberrations, and *T* is the total number of cells.

The necrosis index was calculated by the equation:

(7) NI=(n/T)×100

where *n* is the number of necrotic cells, and *T* is the total number of cells.

The antigenotoxic effect was obtained by the equation [72]:

(8) $$\mathrm{Low}\;\mathrm{CAI}\;(\%)=[({\mathrm{CAI}}_{\mathrm{pc}}-{\mathrm{CAI}}_{s})/({\mathrm{CAI}}_{\mathrm{pc}}-{\mathrm{CAI}}_{\mathrm{nc}})]\times 100$$

where CAI is the chromosomal aberrations, *pc* is the positive control (glyphosate or atrazine), *s* is the extract or fractions of *S. fluminensis* leaves, and *nc* is the negative control (MES).

### 3.9. Statistical Analysis

All experimental data were expressed as the mean ± standard error. Before statistical analysis, the Shapiro–Wilk test and Kolmogorov–Smirnov test were applied to evaluate the normality of the data, and the Brown–Forsythe test or Bartlett’s test was applied to determine the homogeneity of variance. The data for each group were analyzed through one-way ANOVA, and post hoc Dunnett’s test was used to compare significant differences between experimental groups with one negative control (MES) and two positive controls (ATZ and GLY). Statistical analysis was performed using GraphPad Prism 7.0 software (San Diego, CA, USA) with *p* < 0.05 significance levels.

## 4. Conclusions

This is the first study to report the allelopathic, cytotoxic, and antigenotoxic effects of the extract and fractions of *S. fluminensis* leaves. All samples reduced the vigor, germination rate, and GSI of *A. cepa*. In contrast, they did not alter the vigor or viability of *L. sativa*, but they decreased the GSI. Furthermore, all samples inhibited epicotyl and root elongation in *A. cepa* and reduced epicotyl and root growth in *L. sativa*. Thus, these results encourage further studies to evaluate the potential of these samples as pre-emergent herbicides with activity against both monocotyledons and eudicotyledons, warranting future validation in greenhouse and field trials.

All samples, mainly the EE (125 and 250 μg/mL) and HEXF (250 μg/mL), reduced the mitotic index but were not considered cytotoxic. In the genotoxic assay, the samples did not exhibit this activity. In the antigenotoxic assay, the EE (125 and 750 µg/mL), HEXF (750 and 1000 µg/mL), DCMF (125, 250, and 1000 µg/mL), EAF (at all tested concentrations), and HEF (125, 250, and 750 µg/mL) exhibited an antigenotoxic effect after ATZ pretreatment, while the EAF (125 and 500 µg/mL) and HEF (125 µg/mL) demonstrated the potential to reverse genetic damage after GLY pretreatment. Therefore, antigenotoxic compounds from *S. fluminensis* leaf extracts hold promise as allelochemicals in agriculture to mitigate the harmful effects of synthetic herbicides, promoting sustainable agricultural practices. Our findings encourage further studies to assess the effects of this extract and its fractions on environmental safety, crop selectivity, soil persistence, and non-target organisms.

## Figures and Tables

**Table 1:** Seed germination of *Allium cepa* and *Lactuca sativa* treated with the ethanol extract (EE) and fractions (HEXF, DCMF, EAF, and HEF) from *Smilax fluminensis* leaves and positive controls (ATZ and GLY).

	*A. cepa*				*L. sativa*			
	μg/mL	1st Count (%)	Germination Rate (%)	GSI	μg/mL	1st Count (%)	Germination Rate (%)	GSI
NC	0	88 ± 3.77	100 ± 0	8.92 ± 0.44	0	78 ± 5.81	93 ± 2.21	11.19 ± 0.44
ATZ	125	95 ± 2.21	95 ± 2.21	6.96 ± 0.19 ^a^	125	64 ± 8.21	71 ± 10.56 ^a^	8.56 ± 0.93 ^a^
	250	91 ± 5.12	96 ± 1.88	6.83 ± 065 ^a^	250	71 ± 1.15	77 ± 2.21 ^a^	9.50 ± 0.47
	500	87 ± 2.21	88 ± 1.88	6.60 ± 0.30 ^a^	500	67 ± 2.21	73 ± 3.94 ^a^	9.21 ± 0.51 ^a^
	750	89 ± 2.21	90 ± 1.33	6.88 ± 0.17 ^a^	750	66 ± 2.30	74 ± 4.00 ^a^	8.92 ± 0.60 ^a^
	1000	82 ± 4.00	82 ± 4.00 ^a^	7.06 ± 0.39 ^a^	1000	64 ± 4.21	75 ± 3.94 ^a^	10.21 ± 0.80
GLY	125	73 ± 5.45	76 ± 3.26 ^a^	6.05 ± 0.30 ^a^	125	nd	nd	nd
	250	79 ± 4,76	82 ± 5.49 ^a^	5.88 ± 0.69 ^a^	250	nd	nd	nd
	500	44 ± 6.25 ^a^	53 ± 6.36 ^a^	2.96 ± 0.39 ^a^	500	nd	nd	nd
	750	13 ± 1.33 ^a^	20 ± 3.52 ^a^	0.93 ± 0.16 ^a^	750	nd	nd	nd
	1000	3 ± 2.53 ^a^	9 ± 3.09 ^a^	0.32 ± 0.18 ^a^	1000	nd	nd	nd
EE	125	73 ± 4.76 ^b^	92 ± 1.88 ^c^	7.74 ± 0.39 ^c^	125	65 ± 4.37	80 ± 1.88	8.75 ± 0.34 ^a^
	250	73 ± 6.07	94 ± 1.33	7.90 ± 0.44 ^c^	250	69 ± 2.21	81 ± 3.94	9.27 ± 0.46 ^a^
	500	65 ± 5.77 ^abc^	85 ± 13.11 ^c^	6.87 ± 0.80 ^ac^	500	59 ± 3.46 ^a^	76 ± 3.26 ^a^	7.38 ± 0.41 ^ab^
	750	70 ± 6.11 ^bc^	89 ± 2.90 ^c^	7.37 ± 0.33 ^ac^	750	67 ± 1.15	82 ± 7.18	7.89 ± 0.29 ^a^
	1000	65 ± 8.71 ^ac^	94 ± 5.49 ^c^	6.32 ± 0.54 ^ac^	1000	62 ± 7.18	73 ± 9.86 ^a^	6.58 ± 0.82 ^ab^
HEXF	125	57 ± 2.21 ^abc^	83 ± 7.63	5.23 ± 0.37 ^ab^	125	78 ± 6.92	79 ± 7.39	8.94 ± 0.55 ^a^
	250	59 ± 6.07 ^abc^	90 ± 2.98	5.23 ± 0.24 ^a^	250	81 ± 5.77	83 ± 3.94	9.37 ± 0.88 ^a^
	500	69 ± 5.12 ^c^	93 ± 3.94 ^c^	5.58 ± 0.30 ^ac^	500	76 ± 3.26	79 ± 3.46 ^a^	8.64 ± 0,42 ^a^
	750	61 ± 7.86 ^abc^	93 ± 5.12 ^c^	5.89 ± 0.57 ^ac^	750	89 ± 3.46 ^b^	91 ±4.76 ^b^	10.13 ± 0.26
	1000	54 ± 4.00 ^abc^	96 ± 1.88 ^c^	4.84 ± 0.35 ^abc^	1000	88 ± 3.26 ^b^	89 ± 3.46	9.69 ± 0.48
DCMF	125	62 ± 6.66 ^ab^	90 ± 2.98 ^c^	5.67 ± 0.45 ^ab^	125	77 ± 3.94	82 ± 7.18	8.71 ± 0.69 ^a^
	250	56 ± 2.66 ^abc^	81 ± 4.76 ^ab^	5.27 ± 0.19 ^a^	250	76 ± 3.77	83 ± 5.12	8.72 ± 0.44 ^a^
	500	68 ± 5.65 ^ac^	96 ± 3.26 ^c^	6.26 ± 0.51 ^ac^	500	59 ± 6.07 ^a^	72 ± 5.96 ^a^	6.25 ± 0.51 ^ab^
	750	50 ± 6.11 ^abc^	86 ± 6.11 ^c^	4.72 ± 0.43 ^abc^	750	61 ± 9.68	81 ± 3.94	6.61 ± 0.67 ^ab^
	1000	41 ± 6.36 ^abc^	76 ± 8.43 ^ac^	4.29 ± 0.72 ^abc^	1000	41 ± 2.90 ^ab^	55 ± 1.15 ^ab^	4.03 ± 0.18 ^ab^
EAF	125	60 ± 4.98 ^ab^	94 ± 2.98 ^c^	5.70 ± 0.32 ^ab^	125	80 ± 1.88 ^b^	85 ± 2.90	9.32 ± 0.36
	250	53 ± 7.86 ^abc^	84 ± 4.98 ^a^	5.13 ± 0.50 ^ab^	250	85 ± 2.90	89 ± 3.46	9.39 ± 0.33
	500	59 ± 7.86 ^ab^	83 ± 5.77 ^c^	5.45 ± 0.61 ^ac^	500	78 ± 2.98	88 ± 4.98 ^b^	9.19 ± 0.73 ^a^
	750	55 ± 7.39 ^abc^	86 ± 2.98 ^c^	5.35 ± 0.28 ^abc^	750	79 ± 3.94	86 ± 2.98	8.86 ± 0.25 ^a^
	1000	43 ± 8.3 ^abc^	67 ± 7.63 ^ac^	3.98 ± 0.73 ^abc^	1000	71 ± 5.12	78 ± 1.33 ^a^	8.04 ± 0.41 ^a^
HEF	125	69 ± 5.12 ^ab^	97 ± 2.21 ^c^	6.15 ± 0.28 ^a^	125	86 ± 7.18 ^b^	94 ± 4.00 ^b^	10.07 ± 0.79
	250	72 ± 6.53	96 ± 3.26 ^c^	6.41 ± 0.40 ^a^	250	83 ± 5.12	92 ± 4.98 ^b^	10.05 ± 0.49
	500	57 ± 6.07 ^ab^	80 ± 6.53 ^c^	5.29 ± 0.45 ^ac^	500	89 ± 1.15 ^b^	91 ± 2.21 ^b^	11.55 ± 0.62 ^b^
	750	68 ± 4.98 ^abc^	86 ± 8.94 ^c^	6.06 ± 0.65 ^ac^	750	84 ± 4.61 ^b^	85 ± 3.46	10.90 ± 087 ^b^
	1000	76 ± 6.25 ^c^	95 ± 2.21 ^c^	6.65 ± 0.62 ^ac^	1000	84 ± 3.77 ^b^	86 ± 4.42	10.29 ± 1.05

GSI: germination speed index; NC: negative control-(2-(*N*-morpholino) ethane sulfonic acid); ATZ: atrazine; GLY: glyphosate; EE: ethanol extract; HEXF: hexane fraction; DCMF: dichloromethane fraction; EAF: ethyl acetate fraction; HEF: hydroethanol fraction; nd: not determined. Results are mean ± standard error (*n* = 4). ^a^ Statistically significant difference in relation to the NC (*p* < 0.05). ^b^ Statistically significant difference in relation to ATZ, considering samples at the same concentrations (*p* < 0.05). ^c^ Statistically significant difference in relation to GLY, considering samples at the same concentrations (*p* < 0.05).

**Table 2:** Results of genotoxicity tests observed in *Allium cepa* treated with the EE and HEXF, DCMF, EAF, HEF, and positive controls (ATZ and GLY).

Sample	Treatments (μg/mL)	MI	CAI	NI
NC	0	10.33 ± 1.64	3.143 ± 0.08	0.27 ± 0.17
ATZ	125	12.62 ± 2.72	9.81 ± 0.45 ^a^	0.24 ± 0.04
	250	5.57 ± 0.54	13.91 ± 2.66 ^a^	2.14 ± 1.09
	500	5.43 ± 1.25	16.71 ± 1.92 ^a^	0.24 ± 0.09
	750	3.76 ± 0.25	10.67 ± 2.15 ^a^	1.95 ± 1.14
	1000	7.86 ± 0.86	19.24 ± 2.69 ^a^	3.19 ± 1.28
GLY	125	8.05 ± 0.56	9.19 ± 3.28 ^a^	3.29 ± 2.13
	250	4.29 ± 0.38	5.38 ± 1.80	1.48 ± 1.27
	500	14.86 ± 2.08	8.14 ± 0.81	0.05 ± 0.05
	750	23.00 ± 3.07 ^a^	8.33 ± 1.12	0.05 ± 0.05
	1000	6.71 ± 3.60	44.95 ± 2.88 ^a^	26.10 ± 2.72 ^a^
EE	125	3.19 ± 1.04 ^ab^	0.71 ± 0.22 ^bc^	0.00 ± 0.00 ^c^
	250	2.52 ± 0.93 ^a^	0.91 ± 0.13 ^b^	0.00 ± 0.00
	500	3.48 ± 0.29 ^c^	0.71 ± 0.14 ^bc^	0.00 ± 0.00
	750	5.52 ± 2.31 ^c^	1.48 ± 0.60 ^bc^	0.00 ± 0.00
	1000	1.67 ± 0.41	4.14 ± 0.58 ^bc^	0.62 ± 0.62 ^c^
HEXF	125	1.05 ± 0.56 ^bc^	2.57 ± 0.46 ^bc^	0.43 ± 0.43
	250	1.62 ± 0.92 ^a^	1.52 ± 0.21 ^b^	0.38 ± 0.38
	500	1.00 ± 0.79 ^c^	2.52 ± 0.45 ^bc^	0.05 ± 0.05
	750	5.52 ± 2.73 ^c^	3.24 ± 1.63 ^b^	0.00 ± 0.00
	1000	6.62 ± 3.56	3.33 ± 1.05 ^bc^	0.09 ± 0.09 ^c^
DCMF	125	3.10 ± 0.47	2.62 ± 0.39 ^bc^	0.29 ± 0.29 ^c^
	250	5.10 ± 2.11	2.76 ± 0.55 ^b^	0.09 ± 0.09
	500	7.62 ± 2.76	3.33 ± 0.53 ^b^	0.91 ± 0.91
	750	4.81 ± 0.98 ^c^	2.76 ± 0.45 ^bc^	0.05 ± 0.05
	1000	6.38 ± 1.49	3.33 ± 0.27 ^bc^	0.24 ± 0.24 ^c^
EAF	125	3.29 ± 1.61	2.91 ± 0.25 ^bc^	1.52 ± 0.59
	250	5.10 ± 1.45	3.00 ± 0.50 ^b^	0.05 ± 0.05
	500	10.86 ± 1.36	7.05 ± 2.26 ^b^	0.09 ± 0.05
	750	8.05 ± 1.88 ^c^	3.81 ± 0.27 ^b^	0.52 ± 0.52
	1000	12.14 ± 3.25	8.14 ± 1.28 ^bc^	1.38 ± 0.87 ^c^
HEF	125	7.24 ± 0.91	8.19 ± 0.83	0.52 ± 0.29
	250	11.57 ± 2.66 ^c^	6.86 ± 1.43 ^b^	0.62 ± 0.25
	500	9.05 ± 4.93	5.62 ± 1.46 ^b^	0.09 ± 0.09
	750	8.05 ± 4.19 ^c^	6.71 ± 1.87	2.29 ± 1.22
	1000	23.19 ± 11.20	9.10 ± 1.36 ^bc^	1.95 ± 0.99 ^c^

MI: mitotic index; CAI: chromosomal aberration index; NI: necrosis index; NC: negative control (2-(*N*-morpholino) ethane sulfonic acid); ATZ: atrazine; GLY: glyphosate; EE: ethanol extract; HEXF: hexane fraction; DCMF: dichloromethane fraction; EAF: ethyl acetate fraction; HEF: hydroethanol fraction. Results are mean ± standard error (*n* = 3). ^a^ Statistically significant difference in relation to the NC (*p* < 0.05). ^b^ Statistically significant difference in relation to ATZ, considering samples at the same concentrations (*p* < 0.05). ^c^ Statistically significant difference in relation to GLY, considering samples at the same concentrations (*p* < 0.05).

**Table 3:** Results of the antigenotoxicity assay observed in *A. cepa* treated with the EE, HEXF, DCMF, EAF, and HEF previously treated with atrazine and glyphosate.

Atrazine					Glyphosate				
Samples	μg/mL	MI	CAI	Low CAI (%)	Samples	μg/mL	MI	CAI	Low CAI (%)
NC	0	6.71 ± 0.22	2.81 ± 0.25	-	NC	0	2.86 ± 0.30	1.10 ± 0.09	-
ATZ	500	8.81 ± 1.28	5.00 ± 0.62 ^a^	-	GLY	500	3.14 ± 0.86	7.71 ± 1.72 ^a^	-
EE	125	4.71 ± 0.87 ^b^	2.86 ± 0.64 ^b^	97.83	EE	125	2.91 ± 1.34	5.43 ± 0.44 ^a^	34.53
	250	2.52 ± 0.54 ^ab^	3.57 ± 0.80	65.22		250	1.67 ± 0.64	3.38 ± 0.50	65.47
	500	7.71 ± 2.09	3.81 ± 0.52	54.35		500	3.81 ± 2.60	5.43 ± 1.54	34.53
	750	5.67 ± 0.86	3.10 ± 0.75 ^b^	86.96		750	5.62 ± 2.74	10.00 ± 1.95 ^a^	nd
	1000	10.19 ± 0.75 ^a^	4.62 ± 0.53 ^a^	17.39		1000	9.24 ± 0.70 ^ac^	14.14 ± 2.07 ^ac^	nd
HEXF	125	2.95 ± 0.63 ^ab^	3.81 ± 1.06	54.35	HEXF	125	8.71 ± 1.30 ^a^	11.00 ± 1.35 ^a^	nd
	250	6.76 ± 0.37	4.10 ± 1.03	41.30		250	8.95 ± 1.22 ^ac^	13.76 ± 2.75 ^ac^	nd
	500	3.38 ± 0.86 ^b^	4.10 ± 0.47	41.30		500	8.10 ± 1.77	10.48 ± 3.14 ^a^	nd
	750	5.81 ± 0.45	2.19 ± 0.41 ^b^	128.26		750	7.10 ± 0.95	6.95 ± 1.85 ^a^	11.51
	1000	5.95 ± 0.50	2.57 ± 0.46 ^b^	110.87		1000	5.24 ± 1.10	12.33 ± 1.70 ^a^	nd
DCMF	125	5.29 ± 0.64 ^b^	2.00 ± 0.44 ^b^	136.96	DCMF	125	12.57 ± 1.82 ^ac^	8.52 ± 0.31 ^a^	nd
	250	6.76 ± 1.38	2.67 ± 0.33 ^b^	106.52		250	11.14 ± 2.40 ^ac^	5.91 ± 0.83 ^a^	27.34
	500	7.10 ± 0.47	3.86 ± 0.71	52.17		500	11.19 ± 1.67 ^ac^	7.10 ± 2.06 ^a^	9.35
	750	12.62 ± 1.12 ^ab^	4.23 ± 0.05	34.78		750	5.00 ± 1.49	6.24 ± 0.91 ^a^	22.30
	1000	2.86 ± 0.30 ^ab^	2.86 ± 0.22 ^b^	97.83		1000	8.38 ± 2.21	7.95 ± 1.34 ^a^	nd
EAF	125	2.91 ± 0.71 ^ab^	2.19 ± 0.39 ^b^	128.26	EAF	125	11.14 ± 1.70 ^ac^	2.81 ± 0.42 ^c^	74.10
	250	0.91 ± 0.77 ^ab^	2.95 ± 0.45 ^b^	93.48		250	4.81 ± 0.31	4.10 ± 0.55	54.68
	500	0.10 ± 0.09 ^ab^	0.67 ± 0.13 ^ab^	197.83		500	8.86 ± 0.95 ^a^	2.43 ± 0.22 ^c^	79.86
	750	7.52 ± 0.67	2.14 ± 0.08 ^b^	130.43		750	6.43 ± 1.82	3.85 ± 1.37	58.27
	1000	3.38 ± 1.08 ^ab^	2.76 ± 0.53 ^b^	102.17		1000	7.95 ± 2.92	11.71 ± 4.13 ^a^	nd
HEF	125	4.14 ± 0.64 ^b^	3.05 ± 0.45 ^b^	89.13	HEF	125	6.05 ± 2.31	2.38 ± 0.47 ^c^	80.58
	250	2.76 ± 0.17 ^ab^	2.81 ± 0.37 ^b^	100.00		250	11.67 ± 1.48 ^ac^	3.86 ± 0.57	58.27
	500	1.48 ± 0.63 ^ab^	9.76 ± 0.50 ^ab^	nd		500	6.86 ± 0.86	5.81 ± 1.04 ^a^	28.78
	750	1.33 ± 0.54 ^ab^	3.33 ± 0.29 ^b^	76.09		750	5.86 ± 0.87	5.00 ± 0.84	41.01
	1000	1.14 ± 0.30 ^ab^	3.76 ± 0.25	56.52		1000	11.24 ± 1.66 ^ac^	6.81 ± 0.38 ^a^	13.67

MI: mitotic index, CAI: chromosomal aberration index; NC: negative control (2-(*N*-morpholino) ethane sulfonic acid), ATZ: atrazine, GLY: glyphosate EE: ethanol extract, HEXF: hexane fraction, DCMF: dichloromethane fraction, EAF: ethyl acetate fraction, HEF: hydroethanol fraction; nd: not determined. Results are mean ± standard error (*n* = 3). ^a^ Statistically significant difference in relation to the MES (*p* < 0.05). ^b^ Statistically significant difference in relation to ATZ at a concentration of 500 μg/mL (*p* < 0.05). ^c^ Statistically significant difference in relation to GLY at a concentration of 500 μg/mL (*p* < 0.05).

## Data Availability

Data are contained within the article.

## Abbreviations

The following abbreviations were used in this article:

EE	Ethanol extract
ATZ	Atrazine
CAI	Chromosomal aberration index
DCMF	Dichloromethane fraction
EAF	Ethyl acetate fraction
GLY	Glyphosate
GSI	Germination speed index
HEF	Hydroethanol fraction
HEXF	Hexane fraction
HMF	Hydromethanol fraction
IPCS	International Program on Chemical Safety
MI	Mitotic index
NC	Negative control
NI	Necrosis index
NMR	Nuclear magnetic resonance
WHO	World Health Organization
